# Chemoproteomic mapping of human milk oligosaccharide (HMO) interactions in cells†

**DOI:** 10.1039/d2cb00176d

**Published:** 2022-10-18

**Authors:** Abdullah A. Hassan, Jacob M. Wozniak, Zak Vilen, Weichao Li, Appaso Jadhav, Christopher G. Parker, Mia L. Huang

**Affiliations:** Department of Molecular Medicine, Scripps Research, 10550 N Torrey Pines Rd. La Jolla CA 92037 USA miahuang@scripps.edu; Department of Chemistry, Scripps Research, 10550 N Torrey Pines Rd. La Jolla CA 92037 USA cparker@scripps.edu; Skaggs Graduate School of Chemical and Biological Sciences, Scripps Research, 10550 N Torrey Pines Rd. La Jolla CA 92037 USA

## Abstract

Human milk oligosaccharides (HMOs) are a family of unconjugated soluble glycans found in human breast milk that exhibit a myriad of biological activity. While recent studies have uncovered numerous biological functions for HMOs (antimicrobial, anti-inflammatory & probiotic properties), the receptors and protein binding partners involved in these processes are not well characterized. This can be attributed largely in part to the low affinity and transient nature of soluble glycan–protein interactions, precluding the use of traditional characterization techniques to survey binding partners in live cells. Here, we present the use of synthetic photoactivatable HMO probes to capture, enrich and identify HMO protein targets in live cells using mass spectrometry-based chemoproteomics. Following initial validation studies using purified lectins, we profiled the targets of HMO probes in live mouse macrophages. Using this strategy, we mapped hundreds of HMO binding partners across multiple cellular compartments, including many known glycan-binding proteins as well as numerous proteins previously not known to bind glycans. We expect our findings to inform future investigations of the diverse roles of how HMOs may regulate protein function.

## Abstract 2

Human milk oligosaccharides (HMOs) are a family of unconjugated soluble glycans found in human breast milk that exhibit a myriad of biological activity.

## Graphical abstract

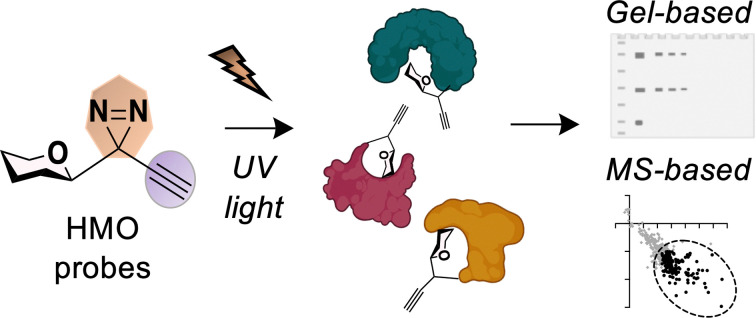

## Introduction

The interactions between glycans and proteins mediate a diverse range of biological processes,^1^ and aberrant glycosylation can result in a myriad of pathologies.^2–4^ Whereas the vast majority of the annotated glycome exists as either lipid- or protein-modified glycoconjugates, “free” (soluble or unconjugated) glycans represent an under-studied fraction of the glycome. From their initial discovery as plant defense signaling molecules^5^ and modulators of host–pathogen interactions,^6^ to the recent discovery of a mammalian disaccharide that induces autoimmunity,^7^ it is becoming increasingly conspicuous that free glycans are important biological regulators.

**Fig. 1:**
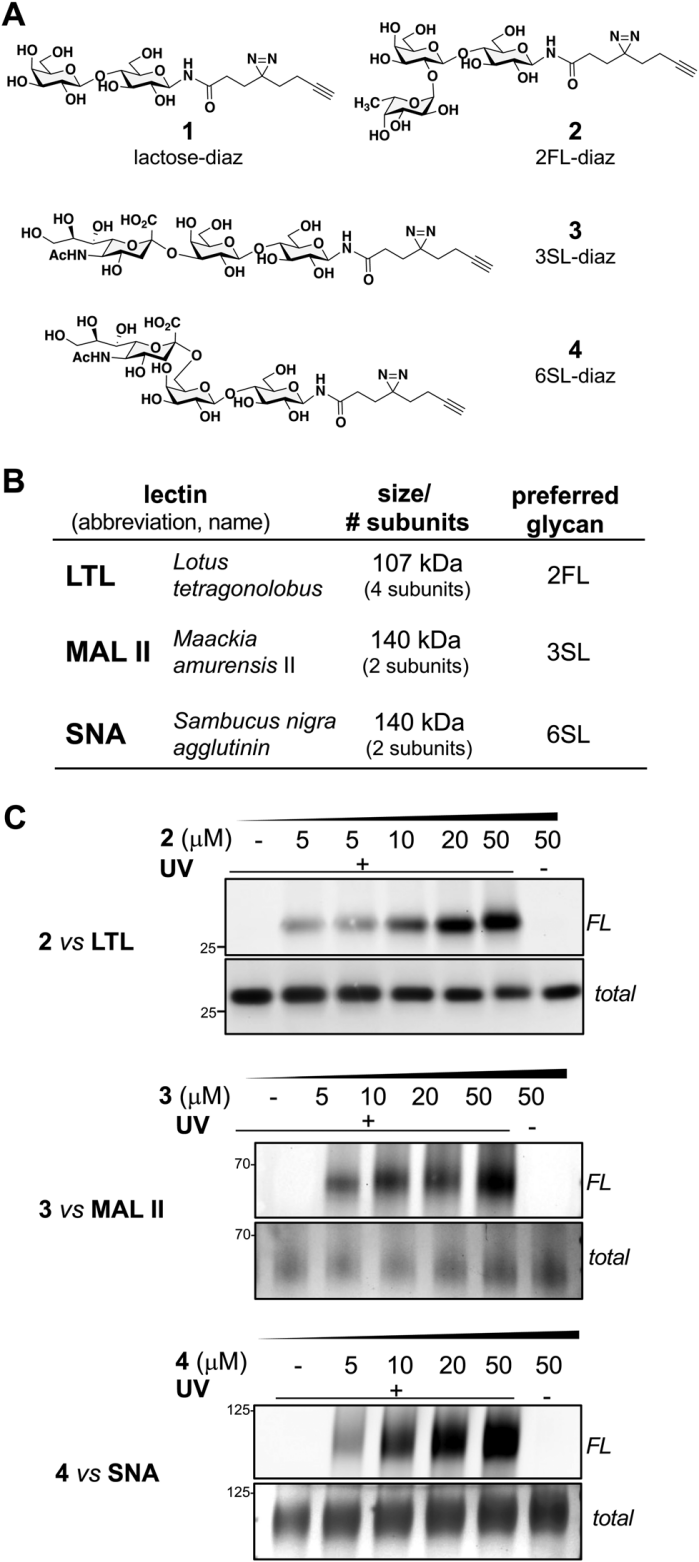
HMO probes bind to purified plant lectins. (A) Chemical structures of HMO probes 1–4. (B) Plant lectins, such as LTL, MALII, and SNA are oligomeric proteins known to exhibit preferences in glycan binding. (C) Analysis of probe engagement with plant lectins. Photo-crosslinking was found to be UV- and dose-dependent.

Human milk oligosaccharides (HMOs) are a family of free glycans that are abundantly found in breast milk.^8,9^ While initially believed to be mere nutritional agents, increasing evidence has highlighted HMOs as important signaling molecules involved in the activation of protein receptors^10^ and the resolution of inflammation.^9,11–13^ Some HMOs also display antimicrobial activity by acting as soluble decoys for pathogenic microbes or through inhibiting biofilm formation.^14,15^ HMOs share a common lactose (Galβ(1-4)Glc) core structure, which is often decorated with additional modifications, such as fucose or sialic acid residues (Fig. 1A). Importantly, such variants can impart vastly different biological activities.^16^ Despite growing evidence demonstrating that HMOs impact diverse biology important to human health, a molecular understanding of the mechanisms and protein partners through which HMOs act remains limited.^12^

Similar to glycoconjugates, the interactions of free glycans with glycan-binding proteins are relatively weak (*K*_D_ ∼ 10^−4^–10^−7^ M), dynamic, and prefer the physiological presentation of proteins in live cells.^17^ These inherent characteristics preclude the use of conventional affinity-based techniques, which require harsh sample preparation procedures. Although several methods have been developed to survey the protein-binding partners of glycoconjugates in live cells, these techniques have not been applied directly to the study of free glycans.^18^

Thus, we hypothesized that photoaffinity labeling could be applied towards the study of HMO interactions in live cells.^19,20^ Photoaffinity labeling is able to capture transient and low affinity interactions, and has been previously combined with metabolic oligosaccharide engineering to profile interactions with glycoconjugates.^21–25^ To enable identification of potential HMO-binding proteins, we chose to utilize a ‘fully-functionalized’ enrichment tag composed of a photoactivatable diazirine group for UV light-induced capture of HMO bound protein targets and an alkyne handle for conjugation to azide-bearing reporter tags *via* copper-mediated azide-alkyne (CuAAC) “click” chemistry.^26,27^ We synthesized four probes composed of the most abundant HMOs in human breast milk (lactose or Lac, 2’fucosyl lactose or 2FL, 3'sialyl lactose or 3SL, and 6'sialyl lactose or 6SL), and first cross-evaluated their engagement with purified glycan-binding lectins *in vitro*, followed by profiling their interactions with live murine macrophages. Using this strategy, we observed that the HMO probes interacted with hundreds of proteins encompassing several intracellular compartments, some of which interacted in a glycan-mediated manner. Among these interactors were galectin-1 and galectin-3, which were validated as 3SL targets in live cells.

## Results and discussion

Using a similar experimental protocol previously reported by the Townsend group to prepare the 2FL probe (2),^28^ the chemical synthesis of our photoactivatable HMO probes commenced with the Kochetkov microwave-assisted conversion of commercially available HMO hemi-acetals into their corresponding amine derivatives,^29,30^ followed by amide bond coupling with a ‘fully-functionalized’ bifunctional diazirine-alkyne acid linker (see ESI†). The probes 1–4 were synthesized in 44% to 53% overall yields (Fig. 1A). The anomeric positions were chosen based on lectin crystallography studies and biological data suggesting that this site would be the most inert position for derivatization.^31–33^

With probes in hand, we evaluated their engagement with plant lectins *in vitro*. Plant lectins, such as LTL, MAL II, and SNA, are commonly used reagents to describe glycan patterns in cells. Plant lectins are known to display a degree of selectivity among various glycans, but they also tolerate a wide array of substitutions (Fig. 1B).^34–36^ Upon incubation of the probes with the plant lectins and UV-light irradiation at 365 nm, the mixtures were reacted with tetramethylrhodamine (TAMRA) azide *via* CuAAC, separated using denaturing SDS-PAGE, and visualized by in-gel fluorescence scanning and silver staining (Fig. 1C).^19^ We observed dose-dependent (0–50 μM) photo cross-linking of probes 2–4 with the lectins (Fig. 1C and Fig. S1, ESI†), and cross-linking was dependent on UV irradiation. Consistent with the multi-subunit nature of plant lectins, we observed several bands in SDS-PAGE corresponding to lectin monomers, dimers, or oligomers (Fig. S1, ESI†).

**Fig. 2:**
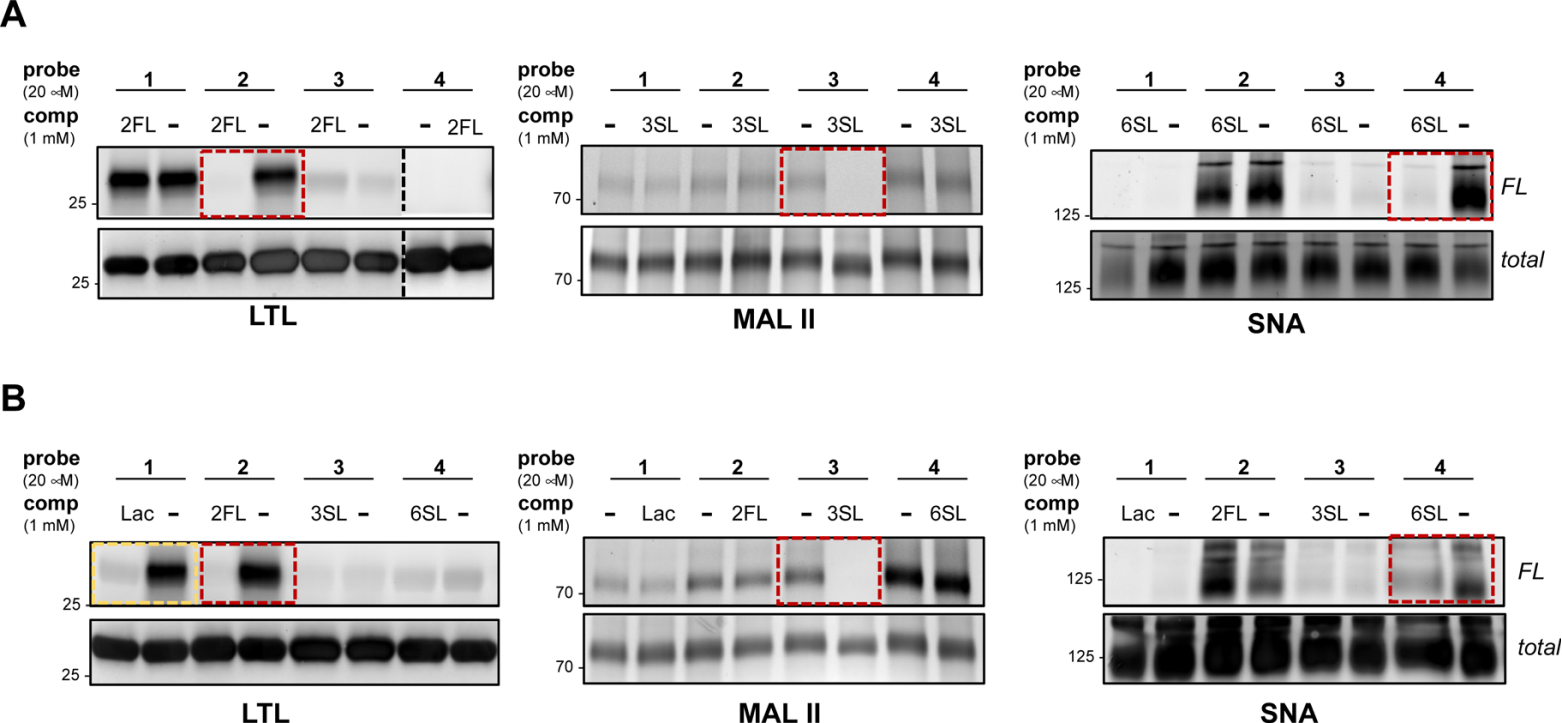
Cross-evaluation of HMO probe binding to recombinant plant lectins and competition experiments. (A) Probes 1–4 differentially engaged LTL, MAL II, or SNA. (B) Excess HMOs (Lac, 2FL, 3SL, or 6SL) can reduce photo-crosslinking. The preferred HMO glycan of each lectin is superior in inhibiting interactions with respective probes (red dashed boxes). We also noted that excess Lac can inhibit crosslinking of LTL with 1 (yellow dashed box).

To further evaluate the selectivity of the probes and determine whether the glycan recognition domains of the lectins were engaged, we performed a cross comparison analysis of different probes against individual lectins and used an array of excess unmodified HMOs as competitors. We observed that LTL was equally captured by probes 1 and 2, and excess 2FL abrogated the interaction with 2 but not with probe 1 (Fig. 2A and Fig. S2A, ESI†). MAL II was engaged by all probes, with probe 4 showing maximum cross-linking. However, only the interaction with probe 3 was competed by excess 3SL. Although cross-linking with SNA was observed with probe 2 and probe 4, only the interaction with probe 4 was reduced by 6SL. We also evaluated the ability of unmodified soluble HMOs (Lac, 2FL, 3SL, 6SL) to reduce photo-crosslinking of the probes (Fig. 2B and Fig. S2B, ESI†), and observed that the preferred glycan of each lectin is superior as a competitor molecule compared to other HMOs. We also note, however, that the interaction of probe 1 in the presence of excess Lac reduced photo-crosslinking with LTL. Overall, these experiments demonstrate that the known preferred glycan ligands are required for successful competition, and that, while probes can cross-react with non-binding lectins, incubation with an excess of the corresponding preferred glycan competes for specific interactions.

**Fig. 3:**
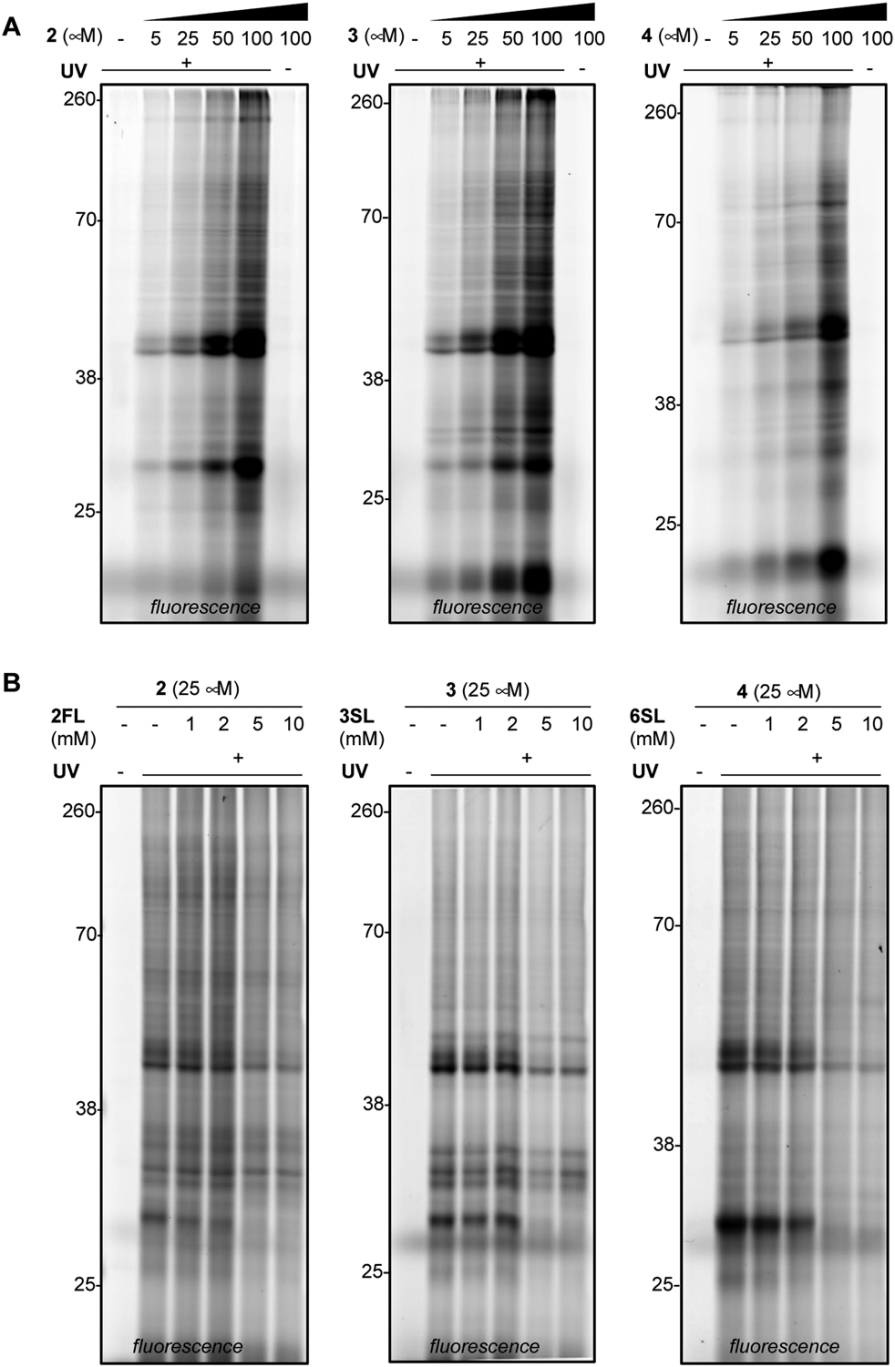
Evaluation of photoactivatable HMO interactions with live mouse macrophages (RAW264.7 cell line). (A) Photoactivatable HMOs engage protein targets in live cells in a dose- and UV-dependent manner. (B) Probe (25 μM) engagement was abrogated with high concentrations of excess unmodified HMOs.

We next evaluated the ability of our probes to engage proteins in live RAW264.7 murine macrophages (Fig. 3A and Fig. S3A, ESI†), as the immunomodulatory activities of HMOs have previously been described in these cells.^11,37^ In addition to the previously reported immunomodulatory roles, RAW264.7 cells are widely used in immunology due to their phenotypic and functional stability.^38^ In brief, cells were treated with each probe, followed by exposure to UV light, harvesting, lysis, coupling of probe-modified proteins to TAMRA-azide and fluorescence visualization. We observed dose-dependent photo-crosslinking with probes 2–4 in cells, with protein targets engaged across a range of molecular weights. Photo-crosslinking was observed to be dependent on the presence of the probe and UV, and probe labeling was significantly blocked by excess soluble HMO (Fig. 3B and Fig. S3B, ESI†), suggesting the selective capture of proteins that recognize the cognate free glycan. We also observed that proteins across multiple cellular compartments, including those that are located within cells were engaged by the probes (Fig. S4A–C, ESI†).

**Fig. 4:**
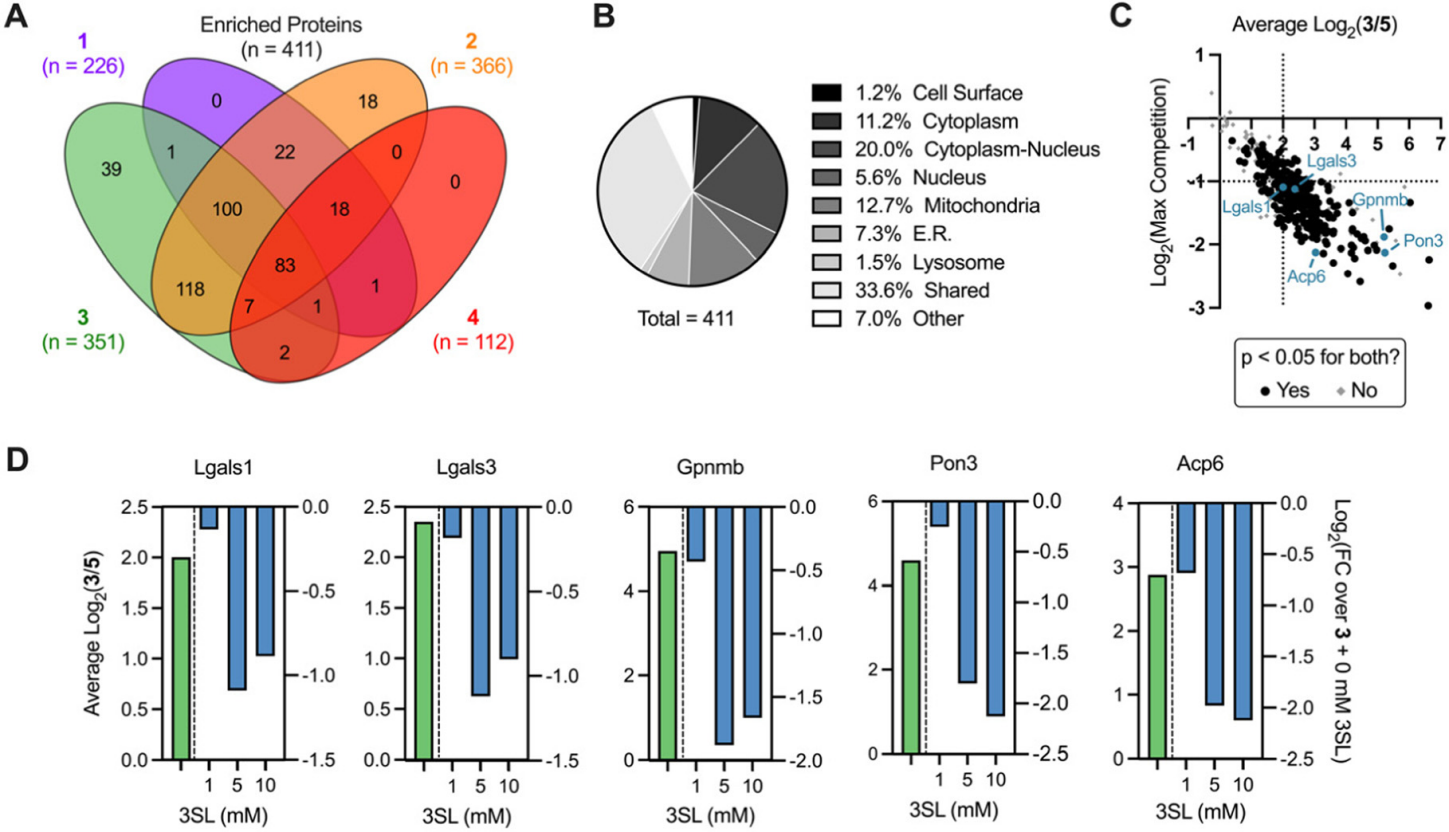
Analysis of HMO probe engagement in live murine macrophages. (A) Venn diagram of overlapping proteins significantly enriched by probes 1, 2, 3 or 4 (>4-fold, *p* < 0.05 relative to probe 5). (B) Cellular compartment analysis of proteins significantly enriched by any probe. (C) Global enrichment-competition plot with selected proteins highlighted. Dotted lines signify cutoffs of >4-fold enrichment (x-axis) and >2-fold competition (y-axis). (D) Focused enrichment (geen bars -competition (blue bars) plots of selected targets (*p* < 0.05 for all enrichments and maximum competition).

We next sought to identify the protein targets of photoactivatable HMOs using quantitative mass spectrometry-based proteomics. Briefly, following probe treatment and photo-crosslinking, we used CuAAC to append a biotin-azide enrichment handle to crosslinked proteins, which were then enriched with streptavidin beads and digested with trypsin. The resulting peptides were subsequently labeled with isobaric tandem mass tags (TMT)^39,40^ to facilitate multiplexed, quantitative comparisons. We also included a control probe (5; see ESI†) to account for possible background interactions dependent on the enrichment tag alone.^19^ Overall, we identified 512 proteins in this experiment (Appendix Table S1, ESI†), observed excellent within replicate correlation and replicates clustered together *via* unbiased hierarchical clustering based on Pearson correlation (Fig. S5A, ESI†). As expected, the median protein abundance (Fig. S5B, ESI†) and number of enriched protein targets (defined as >4-fold, *p* < 0.05 over control probe 5 across two biological replicates; Fig. S4C, ESI†) mirrored the general gel fluorescence intensity for each probe. We identified between 100–350 protein targets for each probe for a total of 411 unique targets across all probes (Fig. 4A). Targets spanned diverse cellular compartments, including the cell surface and intracellular organelles (mitochondria, ER, lysosome, Fig. 4B), consistent with our fractionation and microscopy experiments (Fig. S4A–C, ESI†). While only a fraction (3.4%) of these enriched targets are known to bind carbohydrates, >50% of them are annotated as binding to carbohydrate derivatives or other organic cyclic compounds (Fig. S5D, ESI†). With the exception of probe 4, we identified multiple proteins with robust probe-preferred interactions (Fig. S5E–G, ESI†for probes 1–3, respectively). While probe 4 did not exhibit highly preferred interactions among other probes, it did enrich proteins over one or more of the other probes (Fig. S5H, ESI†), demonstrating selectivity imparted by the different molecular recognition of each glycan moiety.

As probe 3 engaged most of the proteins detected and its cognate free glycan (3SL) displays important immunomodulatory properties,^11,41,42^ we pursued competition experiments to provide additional evidence that the identified targets bind 3SL. In these experiments, cells were pre-treated with excess 3SL prior to incubation with probe 3 plus 3SL and processed for proteomic analysis as described above. We observed excellent correlation between replicates (Fig. S6A, ESI†), and as expected from gel labeling experiments, we observed a strong, dose-dependent reduction of global protein abundances with co-incubation of the 3SL competitor (Fig. S6B, ESI†) and diverse competition profiles (Fig. S6C and D, ESI†).

**Fig. 5:**
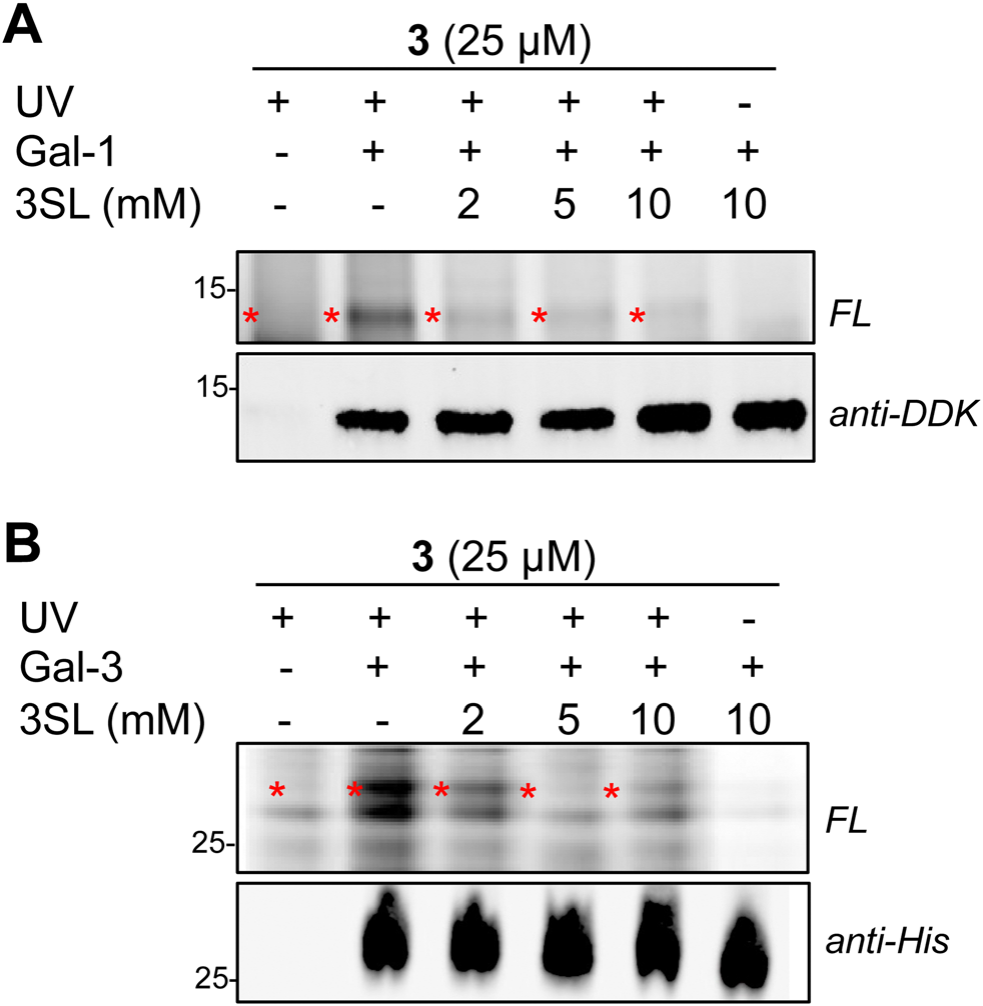
Validation of probe 3 engagement with over-expressed proteins identified as targets in chemoproteomics experiments. (A) Probe 3 captured FLAG(DDK)-tagged-galectin-1 (Gal-1, red asterisk) in HEK293 cells, and photo-crosslinking was reduced by co-incubation with excess 3SL. (B) Probe 3 captured His-tagged galectin-3 (Gal-3, red asterisk) in HEK293 cells, and photo-crosslinking was reduced by co-incubation with excess 3SL.

Overall, we identified 193 unique targets that were both enriched by 3 and competed with coincubation of excess free 3SL (>2-fold, *p* < 0.05 at maximum competition; Appendix Table S2, ESI†). Notably, this list encompasses known glycan-binding proteins including Lgals1 (galectin-1), Lgals3 (galectin-3), and Gpnmb. We also observed proteins that have not been reported to exhibit glycan-binding activities, such as Pon3 (paraoxanase 3) and Acp6 (lysophosphatidic acid phosphatase type 6). (Fig. 4C and D) Intriguingly, both proteins are thought to use small polar molecules (organophosphates, aryl esters and lactones for Pon3) or negatively charged molecules (lysophosphatidic acid for Acp6) as substrates.^43–45^ We selected galectin-1 and galectin-3 as targets for validation studies, which entailed over-expressing FLAG(DDK)-tagged galectin-1 or His-tagged galectin-3 in HEK293T cells (Fig. S7A–C, ESI†). We observed robust photo-crosslinking of galectin-1 (Fig. 5A) or galectin-3 (Fig. 5B) by probe 3 in live cells, which was abrogated by excess 3SL. While galectins (expressed both intra- and extracellularly)^46–48^ are commonly known to bind galactose-terminated glycans, they are also known to tolerate α(2,3)-sialic acid substitutions.^49^ Previous shotgun glycan array studies highlighted that galectins can also bind HMOs and may in some instances be receptors of HMOs.^50^ Together, these data pinpoint high confidence interactors of probe 3 and demonstrate the capture of known glycan binders as well as unexpected proteins with affinity for HMO probes.

## Conclusions

We have demonstrated that fully-functionalized HMO probes can be used to capture and identify potential HMO protein targets in live cells. Our finding that 3SL exhibits maximal interactions with proteins found in murine macrophages is intriguing, given recent findings that it induces transcriptional activation to resolve inflammatory responses, however further investigations are needed to elucidate the underlying molecular mechanisms.^11,41,42^ Overall, these probes augment the existing toolkit to study of glycan–protein interactions in solution and in equilibrium, and we expect the datasets herein to facilitate our understanding HMO biological activity as well as other untapped free glycans.

### Methods

Detailed information regarding the availability and sources of all reagents used as well as experimental procedures used are listed in the ESI.† All raw mass spectrometry proteomics data have been deposited to the ProteomeXchange Consortium *via* the PRIDE partner repository with the dataset identifier PXD035729.

## Conflicts of interest

There are no conflicts to declare

## Supplementary Material

CB-003-D2CB00176D-s001

CB-003-D2CB00176D-s002
