# Supplementary material for: Chemoproteomic mapping of human milk oligosaccharide (HMO) interactions in cells
Source: RSC Chem Biol. 2022 Oct 18;3(12):1369–74. doi: 10.1039/d2cb00176d (PMC9709932; doi:10.1039/d2cb00176d)
Supplement: CB-003-D2CB00176D-s001 [file CB-003-D2CB00176D-s001.pdf]

Electronic Supplementary Material (ESI) for RSC Chemical Biology.
This journal is © The Royal Society of Chemistry 2022

Supporting Information

Chemoproteomic Mapping of Human Milk Oligosaccharide (HMO) Interactions in Cells

Abdullah A. Hassan,[1#] Jacob M. Wozniak,[2#] Zak Vilen,[1,3] Weichao Li,[1,2] Appaso Jadhav,[2] Christopher G. Parker, *[2,3] Mia L. Huang*[1,2,3]

[1] Department of Molecular Medicine, Scripps Research, 10550 N Torrey Pines Rd. La Jolla, CA 92037
[2] Department of Chemistry, Scripps Research, 10550 N Torrey Pines Rd. La Jolla, CA 92037
[3] Skaggs Graduate School of Chemical and Biological Sciences, Scripps Research, 10550 N Torrey Pines Rd. La Jolla, CA 92037

[#] These authors contributed equally to this work
*Correspondence should be directed to cparker@scripps.edu or miahuang@scripps.edu

Table of Contents

Supplementary data
Figure S1. HMO probe engagement with plant lectins in vitro. 2
Figure S2. Cross-comparison of HMO probe engagement with plant lectins and competition with soluble excess HMOs. 3
Figure S3. HMO probe photo-crosslinking with RAW264 cells. 4
Figure S4. HMO probes enter mammalian cells and can engage intracellular targets. 5
Figure S5. Proteomics analysis of HMO probes in RAW264 cells. 6
Figure S6. Proteomics analysis of probe **3** interactions in RAW264 cells and competition 7
Figure S7. HMO probe **3** photo-crosslinks proteins identified from chemoproteomics experiments 8

Materials and general procedures
Chemical reagents and synthesis procedures 9
Biochemical reagents and procedures 11
Photo-crosslinking with recombinant lectins 12
Preparation of proteomics samples 13

Extended compound characterization
HMO probe **1** (^{1}H, ^{13}C NMR) 15
HMO probe **2** (^{1}H, ^{13}C NMR) 16
HMO probe **3** (^{1}H, ^{13}C NMR) 17
HMO probe **4** (^{1}H, ^{13}C NMR) 18
HMO probe **5** (^{1}H, ^{13}C NMR) 19
References 20

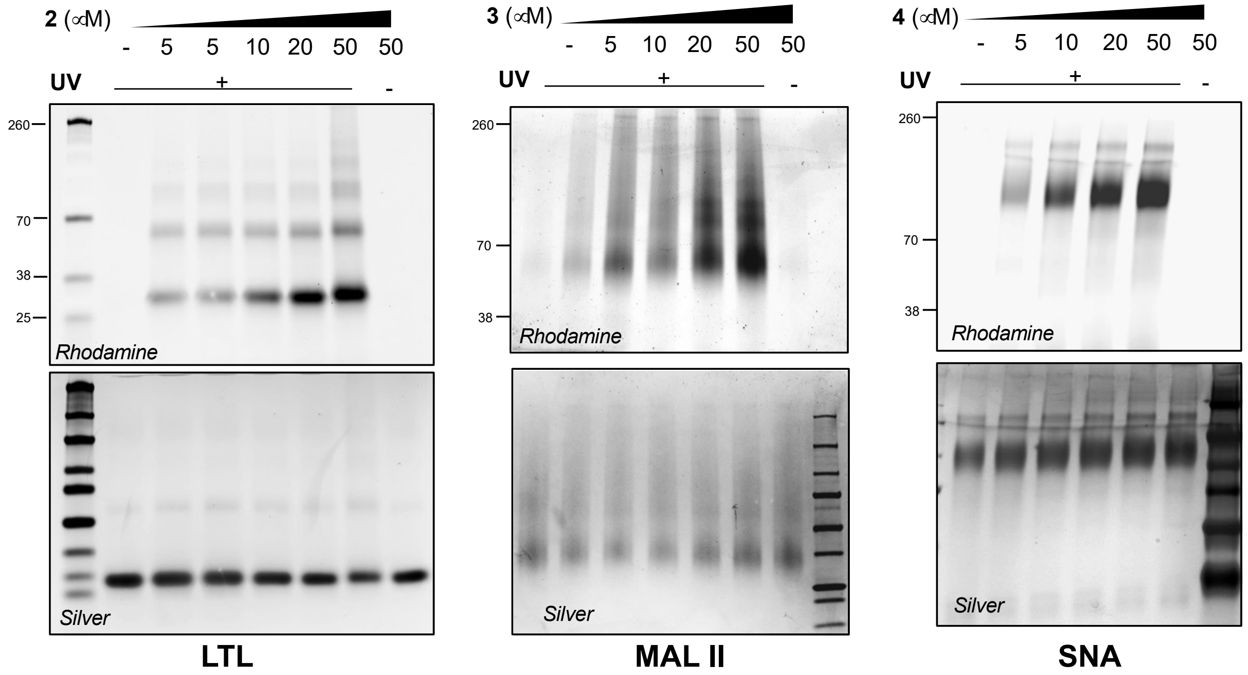

Figure S1. HMO probe engagement with plant lectins in vitro. Full fluorescence and silver stain gel images for main text Figure 1C. Probes **2** – **4** engaged LTL, MALII, and SNA in a UV-dependent and dose-dependent manner (0 - 50 μM). No fluorescence signals were observed in the absence of UV irradiation. Multiple bands correspond to oligomeric states of each lectin, according to main text Figure 1B.

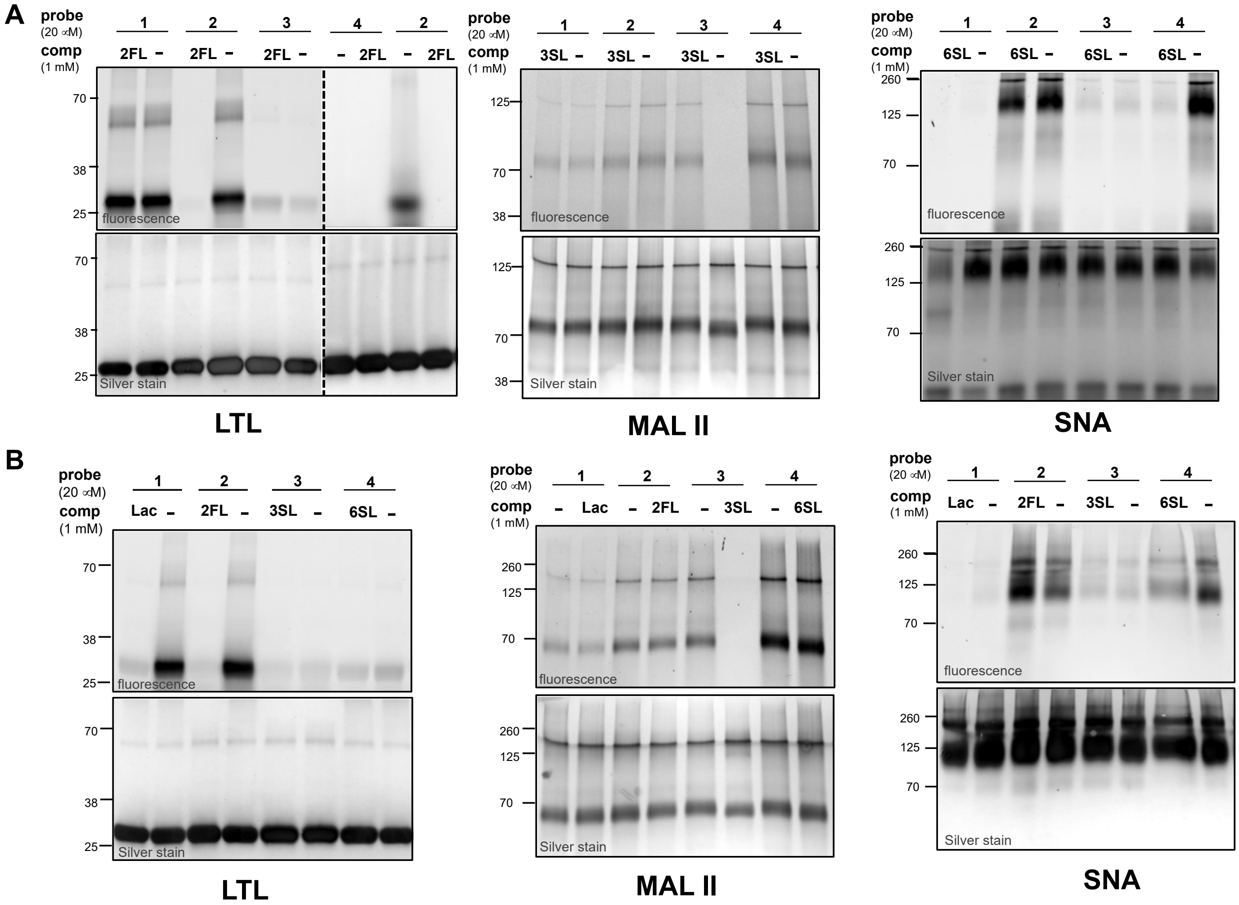

Figure S2. Cross-comparison of HMO probe engagement with plant lectins and competition with soluble excess HMOs. Full fluorescence and silver stain images of main text Figure 2A and 2B. Multiple bands correspond to oligomeric states of each lectin. (**A**) HMO probes can engage multiple lectins, and photo-crosslinking is reduced by the addition of soluble 'preferred' HMO that is known to bind the carbohydrate binding domain of each lectin (2FL for LTL, 3SL for MALII, and 6SL for SNA). (**B**) HMO photo-crosslinking with each lectin is significantly reduced by the preferred HMO.

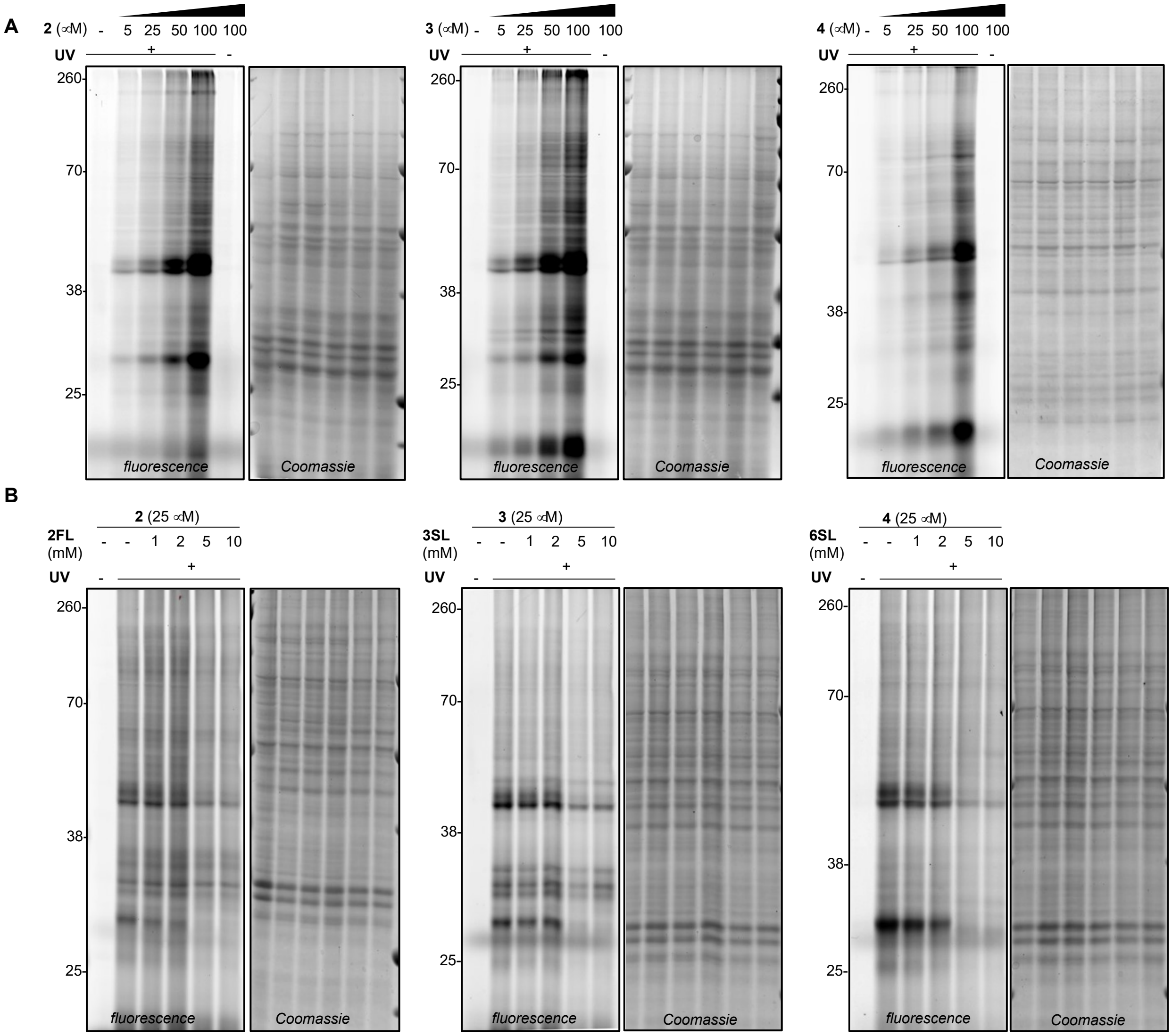

Figure S3. HMO probe photo-crosslinking with RAW264 cells. (**A**) HMO probes label live RAW264.7 cells in a dose-dependent manner. No fluorescence signal is observed in the absence of UV. (**B**) Competition gel profiles of probes **2** – **4.** Photocrosslinking of HMO probes (25 µM) can be competed out by excess 'free' HMOs. Full length gels corresponding to main text Figure 3A and 3B.

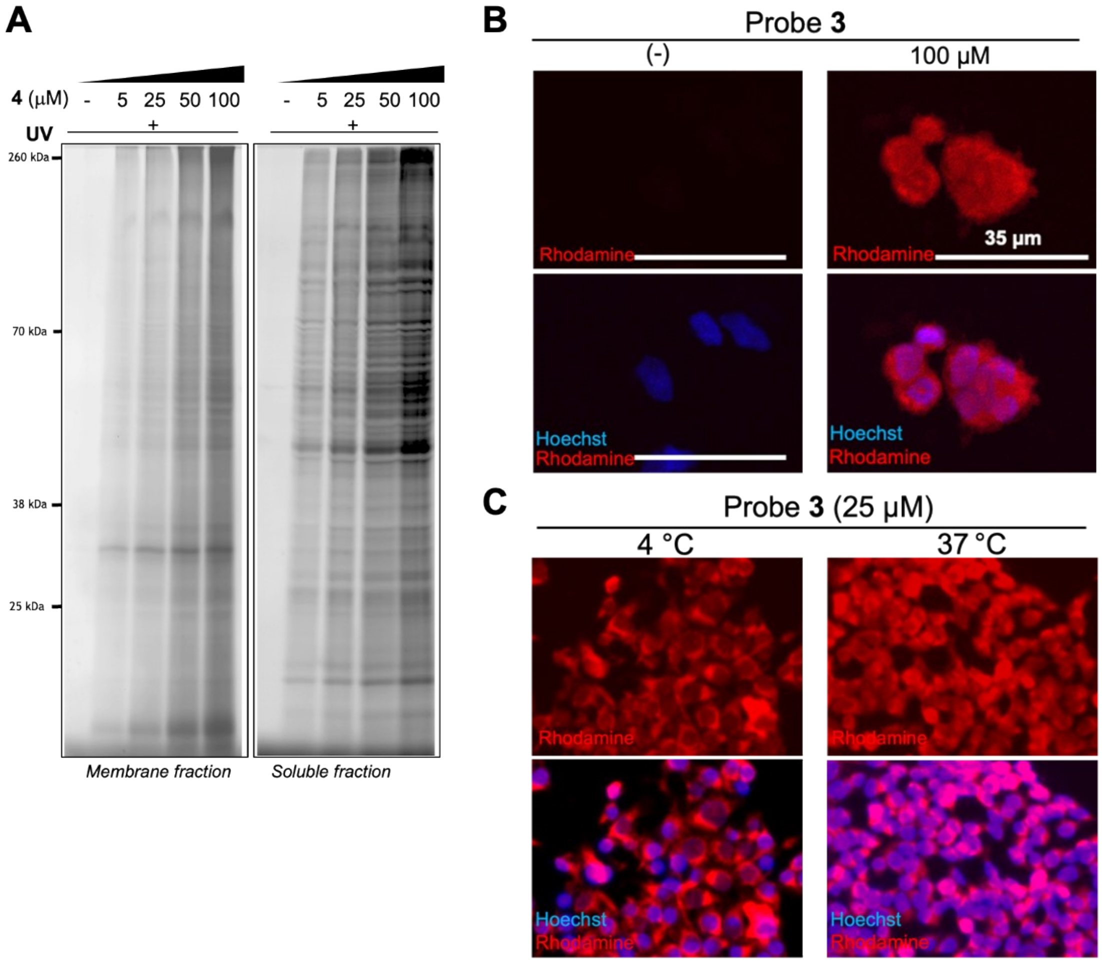

Figure S4. HMO probes enter HEK293 cells and can engage intracellular targets. (**A**) Fluorescent gels of cell lysates following incubation of live cells with probe **4** at 37 °C, lysis, CuAAC conjugation to a TAMRA-azide tag, ultracentrifugation, separation, and separation by SDS-PAGE. Both membrane and soluble fractions show fluorescent photo-crosslinked proteins engaged by probe **4**. (**B**) Confocal microscopy images of live HEK293 cells with probe **4** at 37 °C highlighting cell entry. (**C**) At lower temperatures (4 °C), less fluorescence is observed intracellularly.

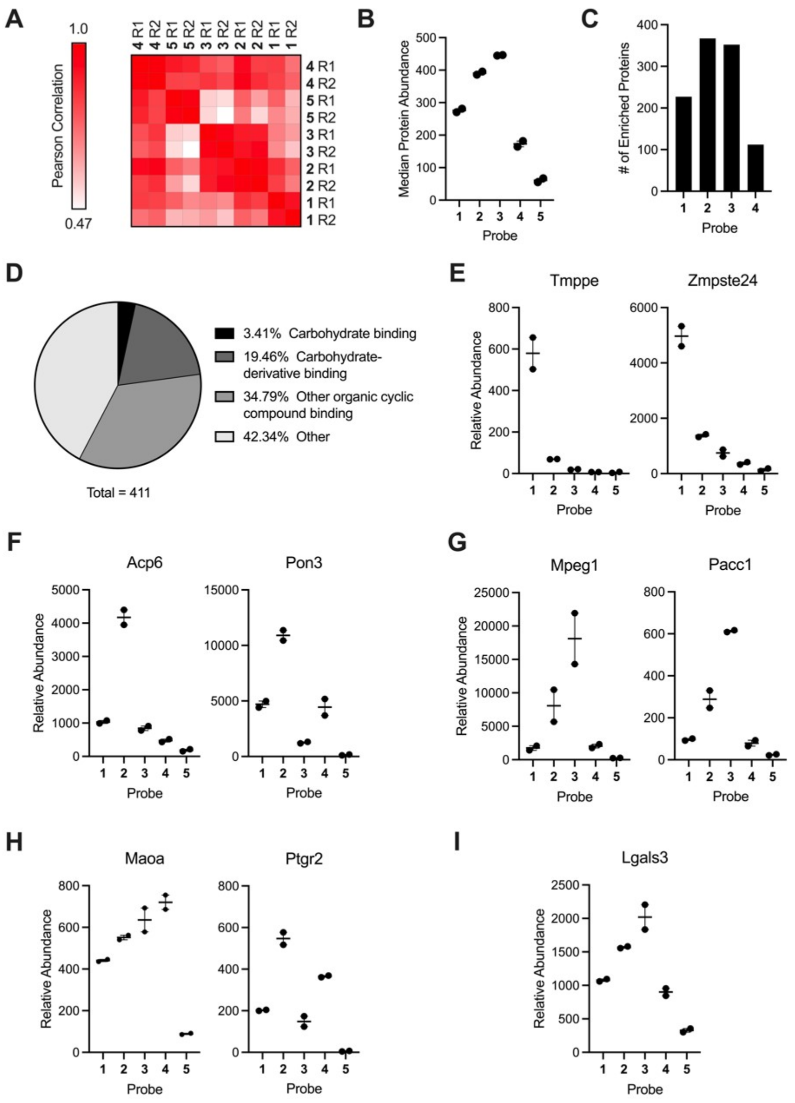

Figure S5. **Proteomics analysis of HMO probe interactions in RAW264 cells**. **(A)** Pearson correlation matrix of quantitative proteomic data from probe comparison experiment. **(B)** Median protein abundance from probe comparison experiment. **(C)** Number of proteins enriched by each probe (>4-fold, p<0.05 relative to probe **5**). **(D)** Carbohydrate and related molecule binding distribution of enriched proteins. **(E)** Examples of probe **1**-preferred proteins. **(F)** Examples of probe **2**-preferred proteins. **(G)** Examples of probe **3**-preferred proteins. **(H)** Examples of probe **4**-preferred proteins. **(I)** Probe comparison data for Galectin-3.

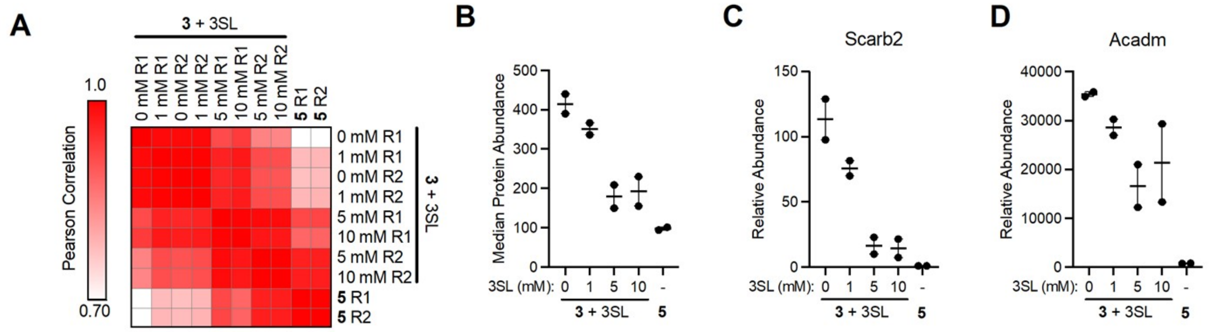

Figure S6. **Proteomics analysis of probe 3 interactions in RAW264 cells and competition**. **(A)** Pearson correlation matrix of quantitative proteomic data from 3SL competition experiment. **(B)** Median protein abundance from 3SL competition experiment. **(C)** Example of a highly competed protein. **(D)** Example of a poorly competed protein.

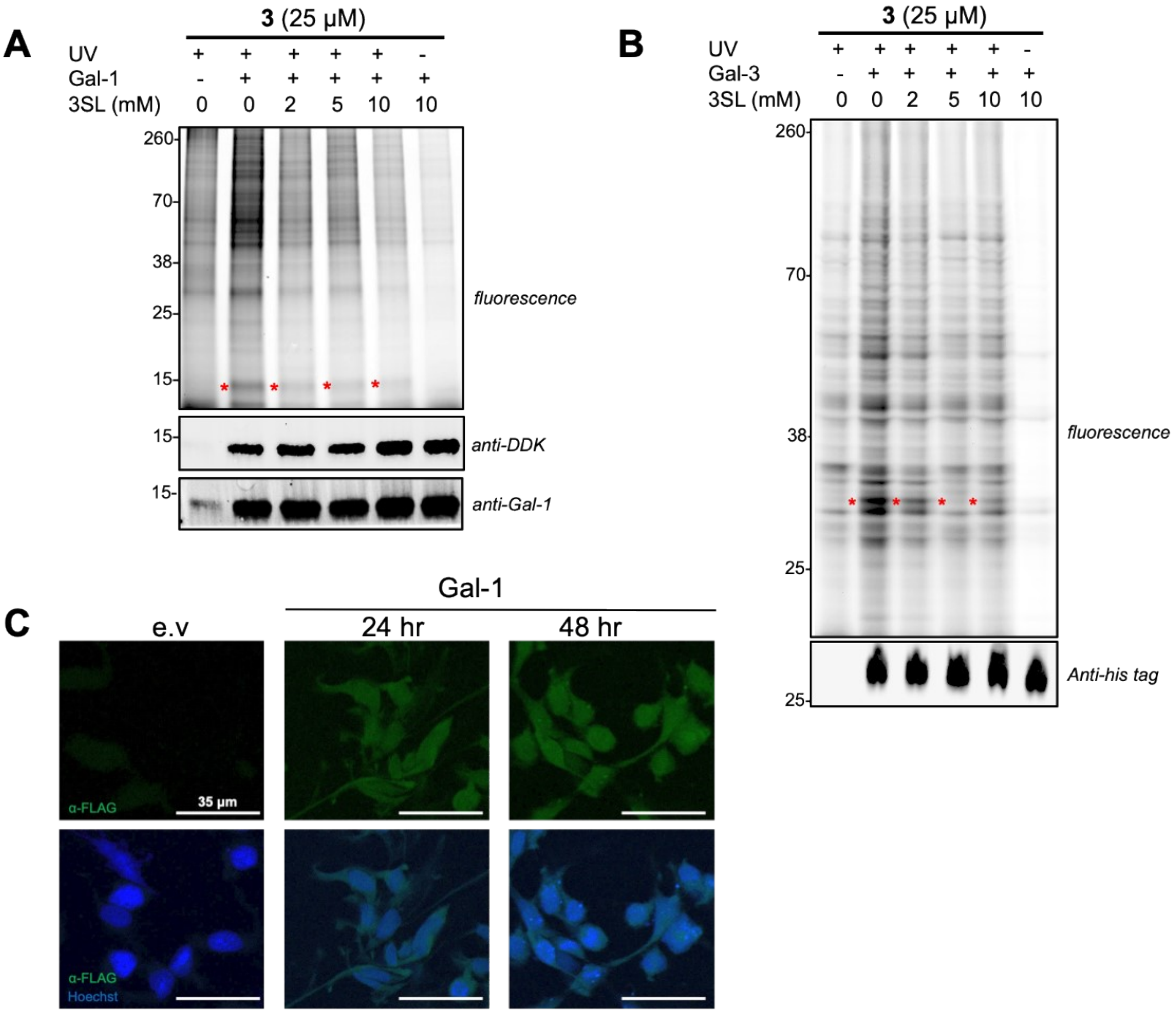

Figure S7. **HMO probe 3 photo-crosslinks proteins identified from chemoproteomics experiments.** HEK293 cells over-expressed with (**A**) DDK-tagged-Gal-1 (red asterisk) or (**B**) His-tagged Gal-3 (red asterisk) and were incubated with **3** (25 µM) in the absence or presence of 3SL (0-10 mM) and photo-crosslinking. Full blots shown for main text Figure 5. (**C**) Over-expressed galectin-1 is present intracellularly. Confocal microscopy images of HEK293 cells over-expressing FLAG-tagged galectin-1 at 24 or 48 hr post-transfection show significant fluorescence (α-FLAG, green) over the empty vector (e.v.) control. Fluorescence signals are located intracellularly.[1-3] Nuclei are stained with Hoechst 33342 (blue).

MATERIALS AND GENERAL PROCEDURES

Chemical reagents

All starting material chemicals were purchased from commercial suppliers (Carbosynth, Sigma Aldrich, Flourochem and Acros) and used without further purification. Unless otherwise stated, all reactions containing air- and moisture sensitive reagents were carried out under an inert atmosphere of nitrogen in oven-dried glassware with magnetic stirring. All reactions were monitored by thin-layer chromatography (TLC) on Merck DC-aluminum plates precoated with silica gel 60 F254. TLC plates were visualized with UV-light (254 nm) and stained with H_2SO_4 (8 %). Silica gel column chromatography was carried out using Davisil silica gel or with automated flash chromatography suite (Biotage SP4 HPFC). ^{1}H NMR (400 or 600 MHz), ^{13}C NMR (125 MHz) spectra were recorded on Bruker AVANCE NEO 400 and 600 MHz instrument at 25 °C in deuterated chloroform ($CDCl_3$), methanol (CD_3OD), or water (D_2O).

Synthesis of HMO probes

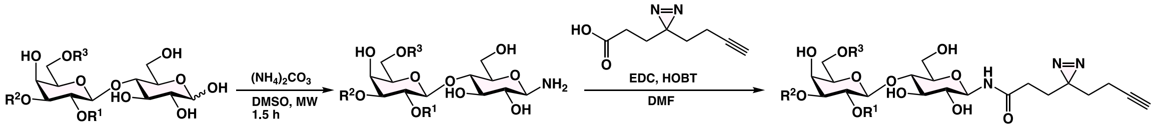

Microwave assisted Kochetkov amination & amidation

Microwave assisted Kochetov amination of HMOs[4-7]. In a Biotage automated microwave vial, HMO reducing sugar (1 eq.) was suspended in anhydrous DMSO (0.1 M) and charged with ammonium carbonate (5 eq.). The tube was sealed and reacted (40 °C, 250 psi, 10 watts, 1.5 hr). Upon competition, the contents were transferred to a round bottom flask and resulting mixture was concentrated *in vacuo* before lyophilization overnight to remove excess ammonia and DMSO. The resulting amorphous off-white glycosyl amines were used without further purification.

Amide coupling procedure. HMO glycosyl amine (1.2 eq) solubilized in anhydrous DMF (0.1 M) was added to a stirred vial containing 3-(3-(but-3-yn-1-yl)-3H-diazirin-3-yl)propanoic acid (1 eq), *N*,*N*-diisopropylethylamine (DIPEA) (1.5 eq), 1-ethyl-3-(3′-dimethylaminopropyl)carbodiimide (EDC-HCl) (1.5 eq) and HOBT (1.5 eq). The resulting mixture was stirred at room temperature overnight. Upon the consumption of the HMO amine, as indicated by TLC analysis, the reaction mixture was diluted with methanol and concentrated under reduced pressure. The crude mixture was purified by flash column chromatography (100% EtOAc →9:1 EtOAc/MeOH, v/v).

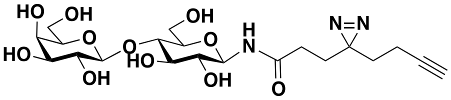

Compound **1** (lactose-diaz) was furnished as an amorphous solid (70 mg, 0.143 mmol, 52%). **^{1}H NMR** (500 MHz, D_2O) δ 5.24 (d, *J* = 5.8 Hz, 1H), 4.47 (d, *J* = 7.5 Hz, 1H), 4.03 – 3.82 (m, 7H), 3.82 – 3.70 (m, 2H), 3.69 – 3.54 (m, 4H), 3.40 – 3.36 (m, 6H), 2.45 – 2.40 (m, 1H), 2.23 (td, *J* = 7.5, 1.7 Hz, 2H), 2.10 (td, *J* = 7.2, 2.6 Hz, 2H), 1.89 – 1.79 (m, 2H), 1.71 (t, *J* = 7.2 Hz, 2H). **^{13}C NMR** (126 MHz, D_2O) δ 177.30, 102.88, 95.74, 91.80, 84.35, 78.44, 75.34, 72.52, 71.40, 71.14, 70.95, 70.09, 69.85, 68.55, 61.02, 59.95, 30.94, 28.55, 28.11, 27.31, 12.41. MS (ESI) calculated for $C_{20}H_{31}N_3NaO_{11}$ (M + Na$^+$) 512.1856, found 512.1861.

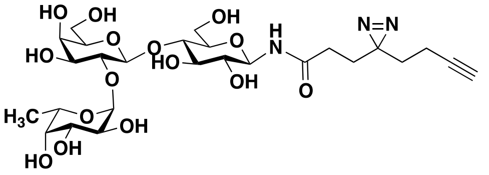

Compound **2** (2'-fucosyllactose-diaz) was afforded as afforded as an off-white amorphous solid (53 mg, 0.083 mmol, 44%). ^{1}**H NMR** (500 MHz, D_2O) δ 5.24 (d, *J* = 3.8 Hz, 1H), 4.65 (d, *J* = 7.9 Hz, 1H), 4.54 (d, *J* = 7.8 Hz, 2H), 3.99 – 3.85 (m, 4H), 3.85 – 3.77 (m, 8H), 3.77 – 3.66 (m, 6H), 3.63 – 3.55 (m, 2H), 2.13 (t, *J* = 7.5 Hz, 4H), 2.09 – 2.05 (m, 2H), 1.78 (t, *J* = 7.5 Hz, 2H), 1.70 (t, *J* = 7.2 Hz, 2H), 1.24 (d, *J* = 6.6 Hz, 3H). ^{13}**C NMR** (126 MHz, D_2O) δ 177.32, 99.33, 95.88, 91.80, 84.34, 76.29, 75.21, 74.29, 73.91, 73.58, 71.66, 69.84, 30.94, 28.55, 28.12, 27.31, 15.26, 15.22, 12.40. MS (ESI) calculated for $C_{26}H_{41}N_3NaO_{15}$ (M + Na^+) 658.2435, found 658.2432

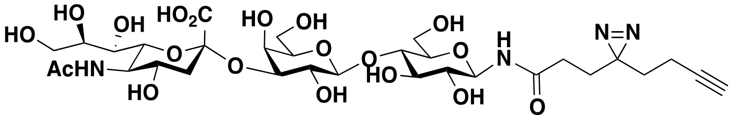

Compound **3** (3'-sialyllactose-diaz) was afforded as a white foam (97 mg, 0.124 mmol, 46%).^{1}**H NMR** (500 MHz, D_2O) δ 4.45 (d, *J* = 7.9 Hz, 1H), 3.91 – 3.71 (m, 7H), 3.71 – 3.45 (m, 10H), 3.21 (t, *J* = 8.5 Hz, 1H), 2.68 (dd, *J* = 12.5, 4.7 Hz, 1H), 2.30 (t, *J* = 2.7 Hz, 1H), 2.15 (t, *J* = 7.4 Hz, 2H), 1.98 (td, *J* = 7.3, 2.8 Hz, 2H), 1.95 (s, 3H), 1.72 (t, *J* = 7.4 Hz, 2H), 1.62 (t, *J* = 7.2 Hz, 2H). ^{13}**C NMR** (126 MHz, D_2O) δ 177.40, 175.01, 173.76, 102.62, 99.73, 95.77, 84.35, 78.18, 75.48, 75.15, 74.79, 74.33, 73.79, 72.88, 71.72, 71.38, 71.14, 69.84, 69.36, 68.29, 68.09, 67.47, 62.59, 61.02, 51.67, 30.94, 28.56, 28.18, 27.34, 22.03, 12.41. MS (ESI) calculated for $C_{31}H_{48}N_4NaO_{19}$ (M + Na^+) 803.2810, found 803.2806.

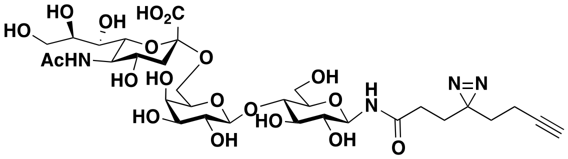

Compound **4** (6'-sialyllactose-diaz) was furnished as a white amorphous solid (45 mg, 0.058 mmol, 53%). ^{1}**H NMR** (500 MHz, D_2O) δ 5.23 (d, *J* = 5.8 Hz, 1H), 4.67 (d, *J* = 8.0 Hz, 1H), 4.54 (d, *J* = 7.9 Hz, 1H), 4.12 (dd, *J* = 9.9, 2.9 Hz, 1H), 4.02 – 3.94 (m, 2H), 3.93 – 3.81 (m, 5H), 3.79 – 3.68 (m, 4H), 3.68 – 3.54 (m, 6H), 3.29 (t, *J* = 8.5 Hz, 1H), 2.77 (dd, *J* = 12.5, 4.7 Hz, 1H), 2.39 (t, *J* = 2.7 Hz, 1H), 2.23 (t, *J* = 7.4 Hz, 2H), 2.07 (dd, *J* = 7.2, 2.7 Hz, 2H), 2.04 (s, 3H), 1.81 (t, *J* = 7.4 Hz, 2H), 1.71 (t, *J* = 7.2 Hz, 2H). ^{13}**C NMR** (126 MHz, D_2O) δ 177.36, 174.91, 173.32, 103.22, 100.18, 84.35, 79.74, 79.63, 74.65, 74.61, 73.67, 72.53, 72.35, 71.73, 70.77, 69.85, 68.50, 68.30, 62.66, 60.26, 51.77, 40.02, 30.94, 28.56, 27.33, 22.05, 12.41. MS: MS (ESI) calculated for $C_{31}H_{47}N_4O_{19}^-$ (M – H^-) 779.2840, found 779.2839.

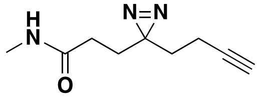

Compound **5** (methyl control probe) was prepared according to previously reported procedures.[8] ^{1}**H NMR** (400 MHz, $CDCl_3$) δ 5.56 (brs, 1H), 2.82 (d, *J* = 2.2 Hz, 2H), 2.08 – 1.98 (m, 3H), 1.94 (m, 2H), 1.90 – 1.83 (m, 2H), 1.66 (t, *J* = 7.4 Hz, 2H). ^{13}**C NMR** (101 MHz, $CDCl_3$) δ 171.84, 82.84, 77.48, 77.16, 76.84, 69.32, 32.53, 30.33, 28.54, 28.00, 26.56, 13.42.

Biochemical reagents

Material name	Vendor	Catalog #	Notes
**MALII** *Maackia amurensis lectin II*	Vector Laboratories Inc.	B-1265-1	Biotinylated; prefers to bind sialic acids in a(2,3) linkage
**SNA** *Sambucus nigra agglutinin*		B-1305-2	Biotinylated; prefers to bind sialic acids in a(2,6) linkage
**LTL** *Lotus tetragonolobus lectin*		B-1325-2	Biotinylated; prefers to bind a-linked fucose glycans
Galectin-1 antibody	R&D Systems	AF1152	Dilution: 1:1000
Galectin-3 antibody	Biolegend	126701	Dilution: 1:1000
Human galectin-1 plasmid	Genscript	ORF Clone: OHu25929D	NM_002305 pcDNA3.1+/C-(K)DYK
Human galectin-3 plasmid	Genscript	n/a	see below
Anti-His antibody-AF647	Biorad	MCA1396A647	Host: mouse; Dilution: 1:2000
Anti-DDK monoclonal antibody	Origene	TA50011-100	Mouse mAb. Clone OTI4C5. Dilution: 1:1000
Donkey anti-mouse IgG AF555	Invitrogen	1984047	Donkey pAb. Dilution: 1:1000
Goat anti-rabbit IgG AF647	Invitrogen	A32733	Goat pAb. Dilution: 1:1000

Cell lines

HEK293T (ATCC# CRL-1573.3) and RAW264.7 (ATCC# TIB-71) cells were maintained in high-glucose Dulbecco's Modified Eagle Medium (DMEM) supplemented with 10% (v/v) fetal bovine serum (FBS, Omega Scientific Inc). Both cell-lines were grown at 37 °C in a humidified 5% CO_2 atmosphere and used between passages 3-15. Cells were passaged twice from cryopreservation before being used in experiments.

HMO probe labeling with lectins for gel-based experiments[8]

The probes and/or corresponding competitors were incubated with the lectins (1 mg/mL) at requisite concentrations for 20 min on ice. Following incubation, photocrosslinking was performed by exposure to 365 nm UV light for 10 min using a Stratgene UV Stratalinker 1800 at 4°C. Non-irradiated conditions were incubated at 4°C for 10 min and covered with aluminum foil. For the CuAAC reaction, 3 µL 'click mixture' comprising of tris[(1-benzyl-1H-1,2,3-triazol-4-yl)methyl]amine (TBTA, 1.7mM in DMSO-tBuOH (1:4 v/v), $CuSO_4$ (50 mM aq.), tris(2-carboxyethyl)phosphine (TCEP, 50 mM aq. – freshly prepared) and tetramethyl rhodamine azide (1.25 mM in DMSO) were added to each well. The reactions were carried out in the absence of light at room temperature for 1 hr. The samples were then mixed with SDS loading buffer (4X Laemmli buffer with 5% β-mercaptoethanol; 5 – 10 µL) without boiling (boiling can degrade the fluorophore) and loaded onto mini SDS-PAGE gels (10% or 4 – 20% gradient, Biorad). The gels were imaged using a Bio-Rad ChemiDoc flatbed fluorescence scanner and the resulting images were processed using Bio-Rad Image Lab software (version 5.2.1). The gels were subsequently stained using Pierce™ Silver Stain Kit (CAT #: 24612).

HMO probe labeling with live cells for gel-based experiments[8]

For gel-based experiments, RAW264.7 cells were grown in 6-well plates to 80% confluency. Upon aspiration of the media, the cells were incubated with fresh serum-free media (1 mL) containing the indicated probe, DMSO vehicle, or with the requisite competitor. For the competition experiments, the indicated competitor was pre-incubated with the cells for 15 min. Then the cells were aspirated and replenished with fresh serum-free media containing the corresponding probe and competitor for a further 30 min. Following incubation, cells were photocrosslinked by exposure to 365 nm UV light for 20 min at 4°C. Subsequently, the cells were harvested in cold DPBS by scraping, centrifuged (1,400 x *g*, 3 min, 4°C), washed with cold DPBS (2X) and then aspirated. Upon sonication (Branson Sonifier probe sonicator: 10 pulses, 30% duty cycle, output setting = 4) of cell pellets in cold DPBS (200 – 500 µL), protein concentration of the samples was determined using DC Protein Assay (Bio-Rad) and normalized to 1.5 mg/mL. CuAAC reactions to conjugate fluorophores to the mixture were carried out as above, using 6 µL of 'click mixture,' and occasional vortexing. The samples (30 µg) were then mixed with SDS loading buffer (25 µL) and resolved by SDS-PAGE (10% acrylamide) made in-house and visualized by in-gel fluorescence on a Bio-Rad ChemiDoc MP imaging system. The resulting images were processed using Bio-Rad Image Lab software (version 5.2.1).

Imaging

HEK293T cells were seeded into 24-well plastic dishes with German glass cover slip inserted inside. Upon HMO probe photocrosslinking or transfection, cells were washed (2 x 400 µL), aspirated and fixed with 4% paraformaldehyde (400 µL) in PBS (10 min, RT). After washing with PBS (2 x 400 µL), flag or histidine-tagged proteins were probed with anti-flag (cat # TA50011-100) or anti-his (cat # MCA1396A647) antibodies in PBST (1 h, RT). Hoescht 3342 (1 µg/mL, 10 min, RT) was used to stain the nuclei. For the click The German glass cover slips were subsequently removed and mounted onto glass slides for visualization. Fluorescence microscopy experiments were performed on EVOS M5000 (ThermoFisher). Confocal microscopic Images were collected on a Zeiss LSM 780 confocal microscope with a Plan-Apo 20X objective (NA 0.8). Pixel size was 0.124 µm. The microscope was adjusted to image at 1 airy unit. Hoechst images were collected with a 405 nm laser at 2% power with filters set to 410 nm – 550 nm. Rhodamine and α-FLAG images were collected with a 561 nm laser at 2% power with filters set to 566 nm –

697 nm. Images were processed using Fiji software. Confocal microscopy was performed at the Core Microscopy facility at Scripps Research.

HMO probe labeling with live cells for mass spectrometry-based experiments

RAW 264.7 cells (~90% confluent) were incubated with HMO probes (25 µM, in serum-free media, 30 min), irradiated (365 nm, 20 min in a cold room, 4 °C), and harvested as before. For competition experiments, cells were pre-treated with respective competitors for 15 min before probe incubation. Cells were lysed as before, and protein concentration was normalized to 2 mg/mL. CuAAC was performed by adding 55 µL of 'click mixture' with biotin-PEG4-azide (Chempep, 10 mM in DMSO) instead of tetramethyl rhodamine azide. To remove excess "click" reagents, proteins were precipitated using 2 mL ice-cold methanol:chloroform (4:1) followed by 1 mL ice-cold DPBS. Precipitated proteins were pelleted by centrifugation and washed 2X with 2 ml methanol:chloroform (4:1). Protein pellets were solubilized (500 µL, 6 M urea/0.2% SDS in DPBS) and reduced by pre-mixing TCEP (200 mM in DPBS) with K_2CO_3 (600 mM in DPBS) and adding 50 µL of the resulting solution to each sample. Protein reduction was carried out at 37 °C for 30 min. Proteins were then alkylated with the addition of iodoacetamide (IAA, 70 µL of a 400 mM solution in DPBS) to each sample and incubating in the dark at RT for 30 min. Following alkylation, samples were diluted to 5.5 mL with DPBS and 130 µL of 10% SDS was added to a final SDS concentration of ~0.2%. Streptavidin agarose beads (Pierce; 50% slurry) were washed 3X with DPBS and 100 µL was added to each sample. Proteins were enriched for 1.5 hr at RT while rotating. Protein-bound beads were centrifuged and washed with 5 ml of the following: 1X with 0.2% SDS in DPBS, 2X with DPBS and 1X with 100 mM triethylammonium bicarbonate (TEAB) in H_2O before transferring them to Protein LoBind tubes (Eppendorf) using 100 mM TEAB in H_2O. Next, 200 µl of a trypsin master mix (2 µg trypsin, 1 mM $CaCl_2$, 100 mM TEAB in H_2O) was added to each sample and the enriched proteins were digested overnight at 37 °C while shaking. Digested peptides were separated from streptavidin beads by centrifugation and the supernatant was retained for downstream processing. The beads were washed once with 100 µL 100 mM TEAB in H_2O, which was pooled with the previous supernatant. Digested peptides were dried then resuspended in 100 mM TEAB in 30% acetonitrile/70% H_2O for tandem-mass-tag (TMT; Thermo Fisher Scientific) labeling according to manufacturer instructions. TMT labeled peptides were dried, resuspended in 60 µL 0.1% trifluoroacetic acid and pooled before desalting. Labeled peptides were desalted using the Pierce™ High pH Reversed-Phase Peptide Fractionation Kit (Thermo), eluted with 300 µL of the following solutions (% acetonitrile in 0.1% TEA; Elution (E) 1: 20%, E2: 40%, E3: 60%, E4: 95%) then pooled prior to LC-MS analysis.

LC-MS Analysis

Samples were resuspended in 65 µL buffer A (0.1% formic acid in H_2O) and 10 µl was loaded onto the analytical column with 2% buffer B (0.1% formic acid in acetonitrile) for 10 min. Peptides were eluted with a gradient of 2-25% buffer B (155 min), 25-45% buffer B (10 min), 45-95% buffer B (5 min), 95% buffer B (2 min), 95-2% buffer B (1 min), 2% buffer B (2 min), 2-95% buffer B (1 min), 95% buffer B (2 min), 95-2% buffer B (1 min), and 2% buffer B (11 min) (200 min total). Eluted peptides were detected using either a Thermo Fisher Orbitrap Fusion and Fusion Lumos mass spectrometer with 2000 V applied to the column and a 3 s cycle time. Scan settings were as follows. MS1 scans – Orbitrap mass analyzer, 375 - 1500 m/z scan range, 120,000 resolution, 1 x 10^6 AGC target, 50 ms maximum injection time, 20 sec dynamic exclusion. Peptide isolation and fragmentation – 0.7 m/z quadrupole isolation window and 30% collision-induced dissociation (CID) energy. MS2 scans – ion trap mass analyzer, rapid scan rate, 1.8 x 10^4 AGC target, 120 ms maximum injection time. Synchronous precursor selection (SPS)[9] and TMT fragmentation – 2 m/z isolation window, 10 SPS ions, 65% higher energy collision dissociation (HCD) energy. MS3

scans – Orbitrap mass analyzer, 100 - 500 m/z scan range, 50,000 resolution, 1.5 x 10^5 AGC target, 120 ms maximum injection time.

MS Data Processing and Analysis

Proteomic data were analyzed using Proteome Discoverer 2.3 (Thermo). Peptide sequences were matched to experimental spectra using the SEQUEST-HT algorithm[10]. The MS1 tolerance was set to 10 ppm and the MS2 tolerance was set to 0.6 Da. Data were searched with full trypsin specificity and a maximum of two missed cleavages. Oxidation of methionine (+15.995) was specified as a variable modification and carbamidomethylation of cysteine (+57.02146) and TMT tags (+229.163) of lysine and peptide N-termini were specified as static modifications. For all experiments, data were searched against the full *Mus musculus* proteome database (Uniprot: downloaded 11/2020; 55,432 sequences) using a false discovery rate of 1% at the protein and peptide level[11, 12]. TMT tags were quantified at the MS3 level with a tolerance of 20 ppm. Only PSMs with an average Signal:Noise (S:N) of >10 and >65% of SPS ions matched to the identified peptide sequence were retained. Processed proteomic data were exported from Proteome Discoverer and further processed and analyzed using Microsoft Excel and GraphPad Prism. Mass spectrometry datasets are deposited on ProteomeXchange.

Transient overexpression in HEK293T cells

HEK293T cells (50-60% confluency) were seeded overnight before the addition of DNA pre-complexed with Fugene HD® (1:3, 30 μg:90 μL) to the media. Following 48 h incubation, the transfected HEK293T cells were subsequently trypsinized and seeded into 6-well plates and allowed to adhere for 12 hrs. Upon adherence to the cell plates, the overexpressed HEK293T cells were treated similarly as above.

Overexpression plasmids

Plasmids were either purchased or generated by Genscript USA, Inc. A DDK(FLAG)-tagged human galectin-1 (NM_002305) ORF clone was purchased. An N-terminal poly-histidine-tagged full-length human galectin-3 NP_002297.2) over-expression plasmid was generated (Genscript) by cloning into the PstI/BamHI restriction sites of the pIRES2-AcGFP1 vector (Takara). Plasmids were transformed into DH10B *E. coli* for amplification and purified by maxiprep (ZymoPURE II Plasmid Maxiprep Kit, Cat. # D4203).

NMR Spectra

^{1}H NMR (500 MHz, D_2O) of compound **1**

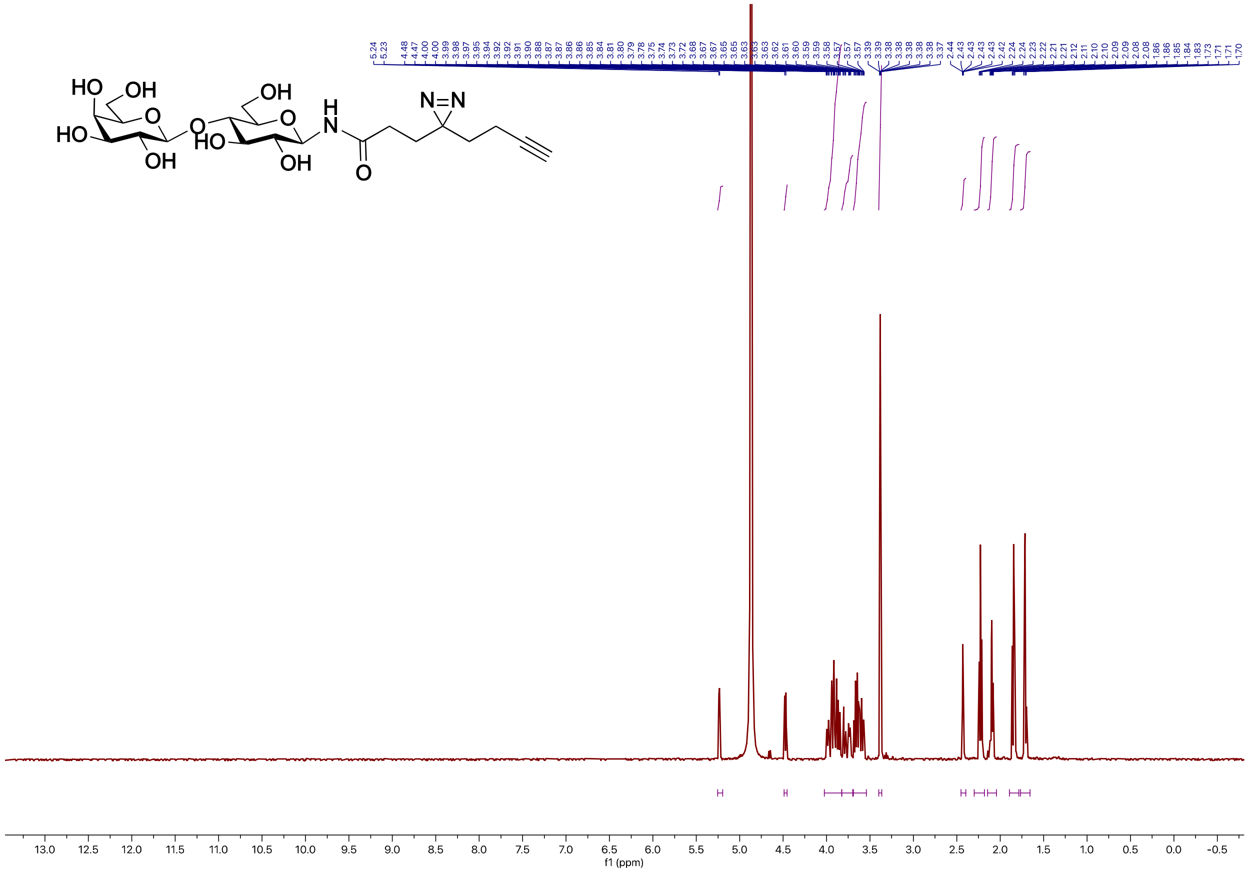

^{13}C NMR (126 MHz, D_2O) of compound **1**

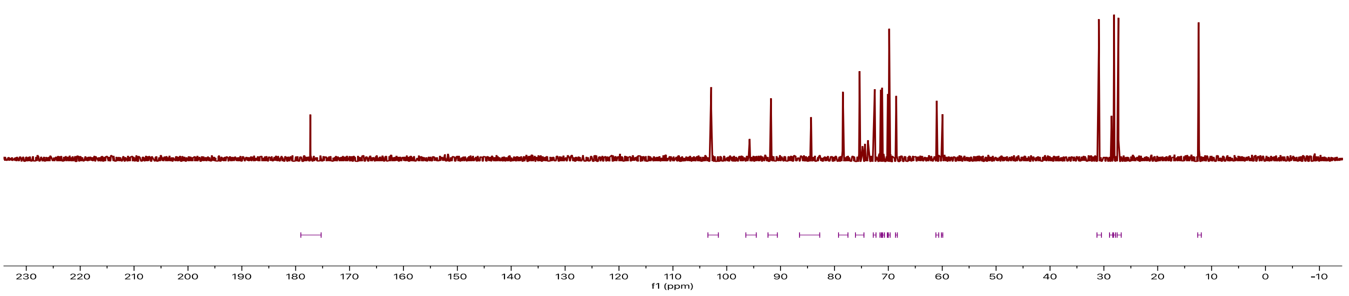

^{1}H NMR (500 MHz, D_2O) of compound **2**

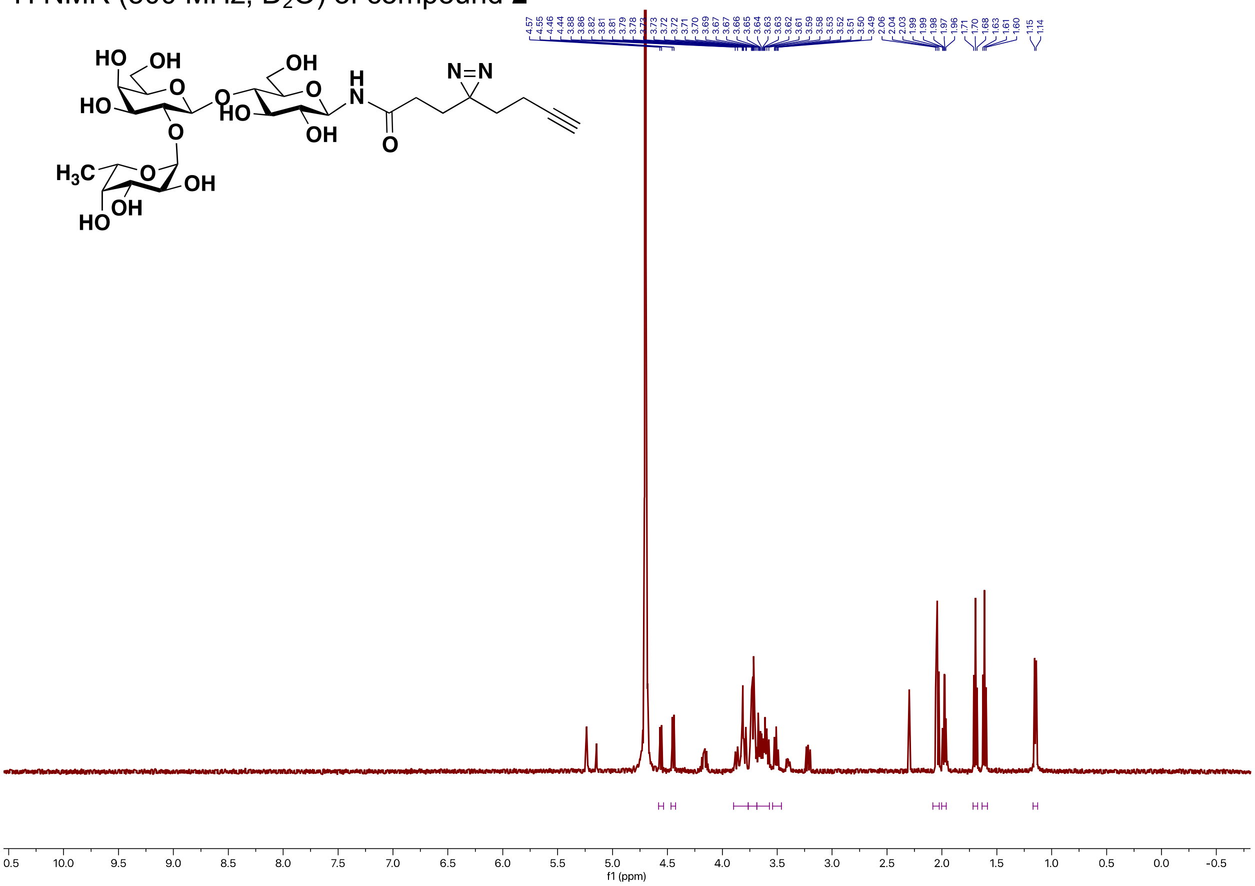

^{13}C NMR (126 MHz, D_2O) of compound **2**

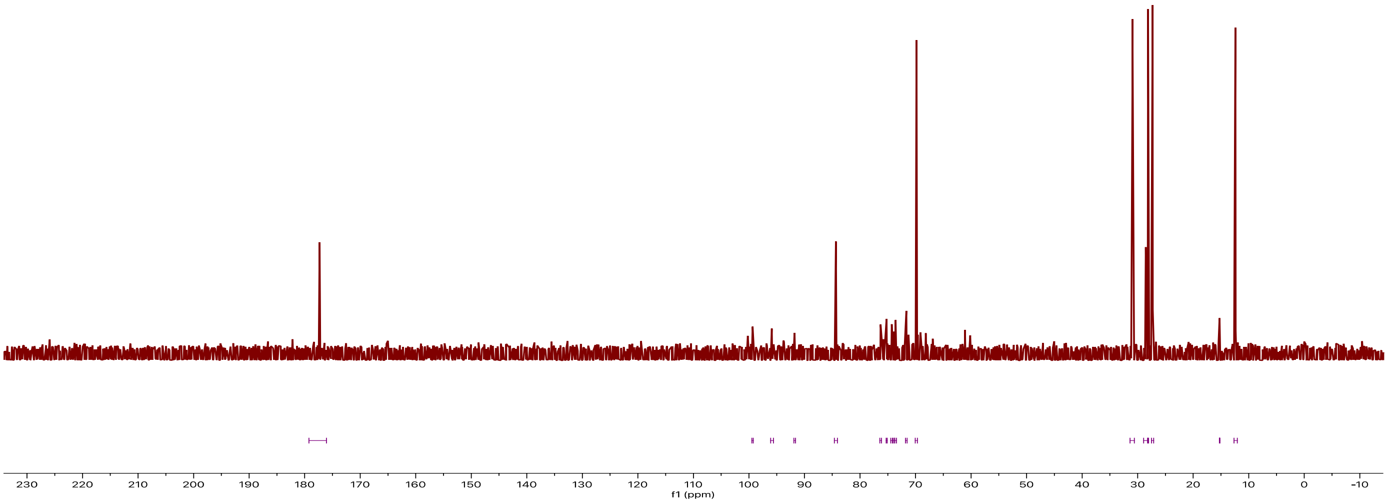

^{1}H NMR (500 MHz, D_2O) of compound **3**

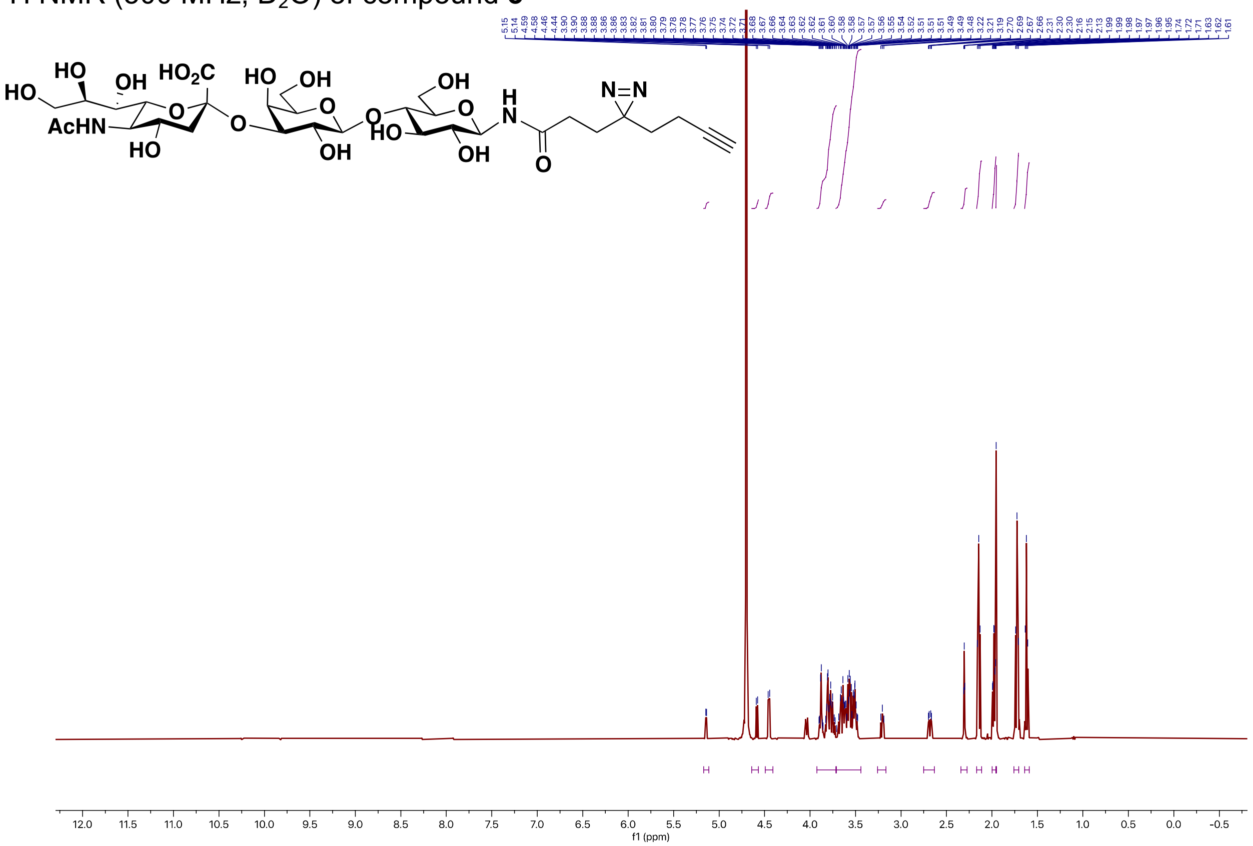

^{13}C NMR (126 MHz, D_2O) of compound **3**

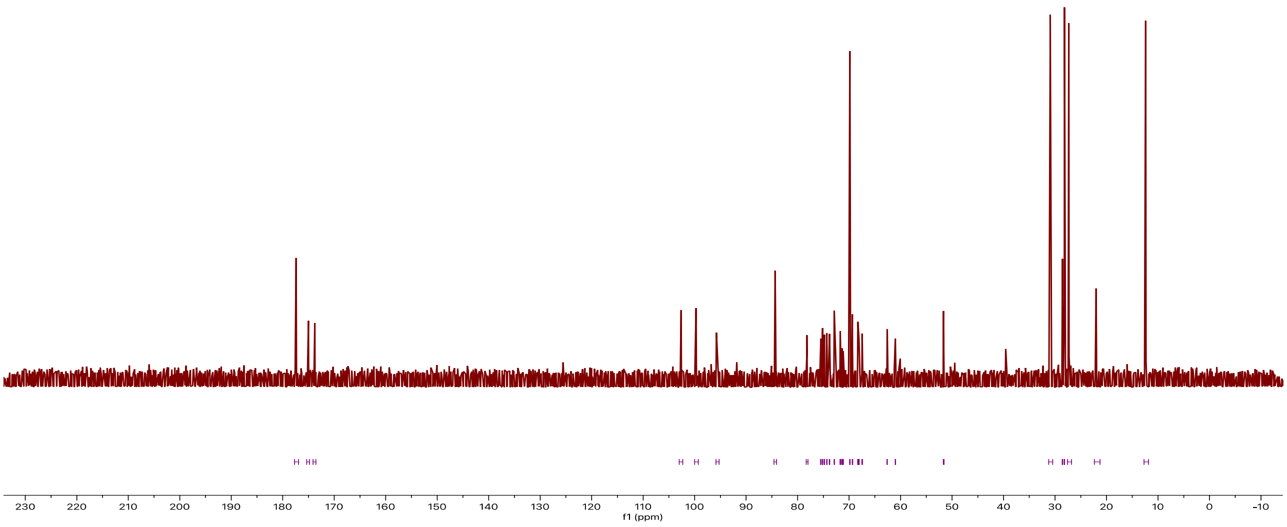

^{1}H NMR (500 MHz, D_2O) of compound **4**

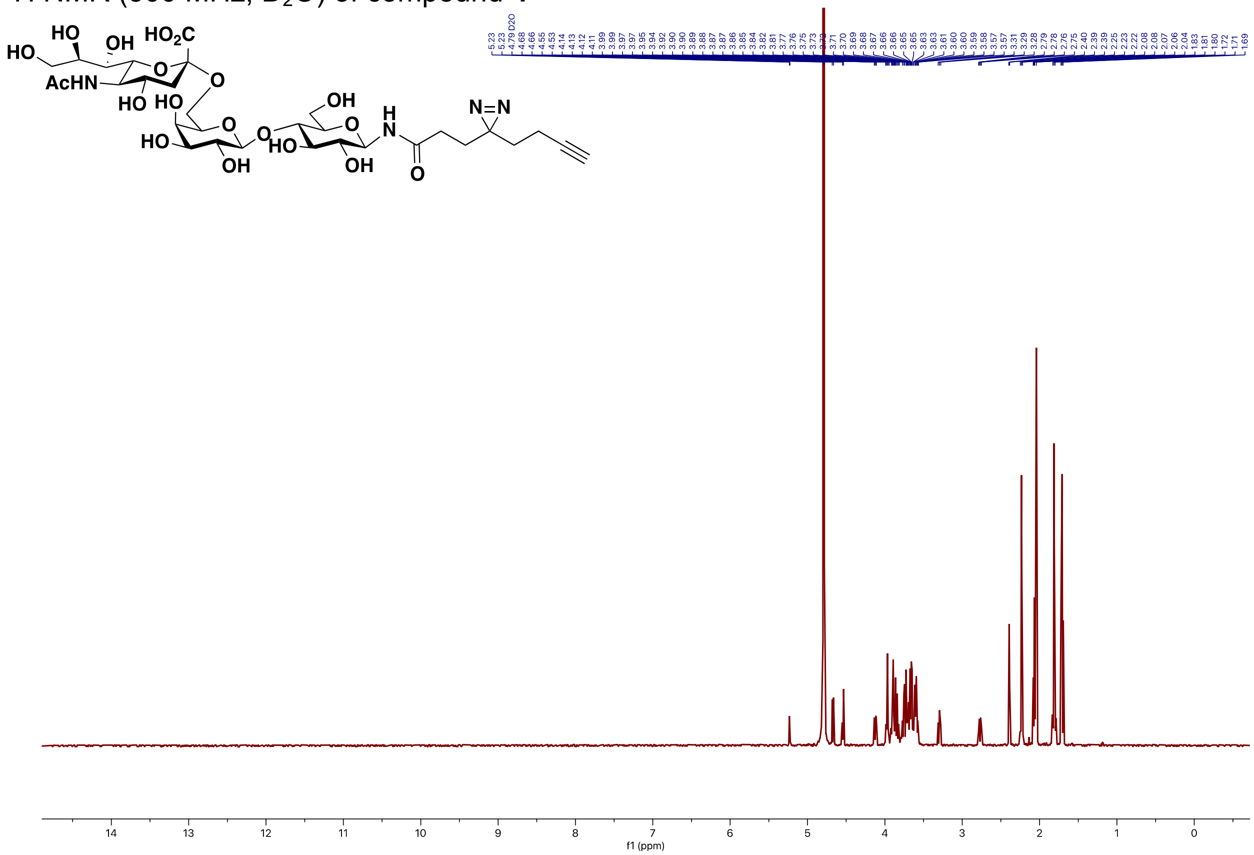

^{13}C NMR (126 MHz, D_2O) of compound **4**

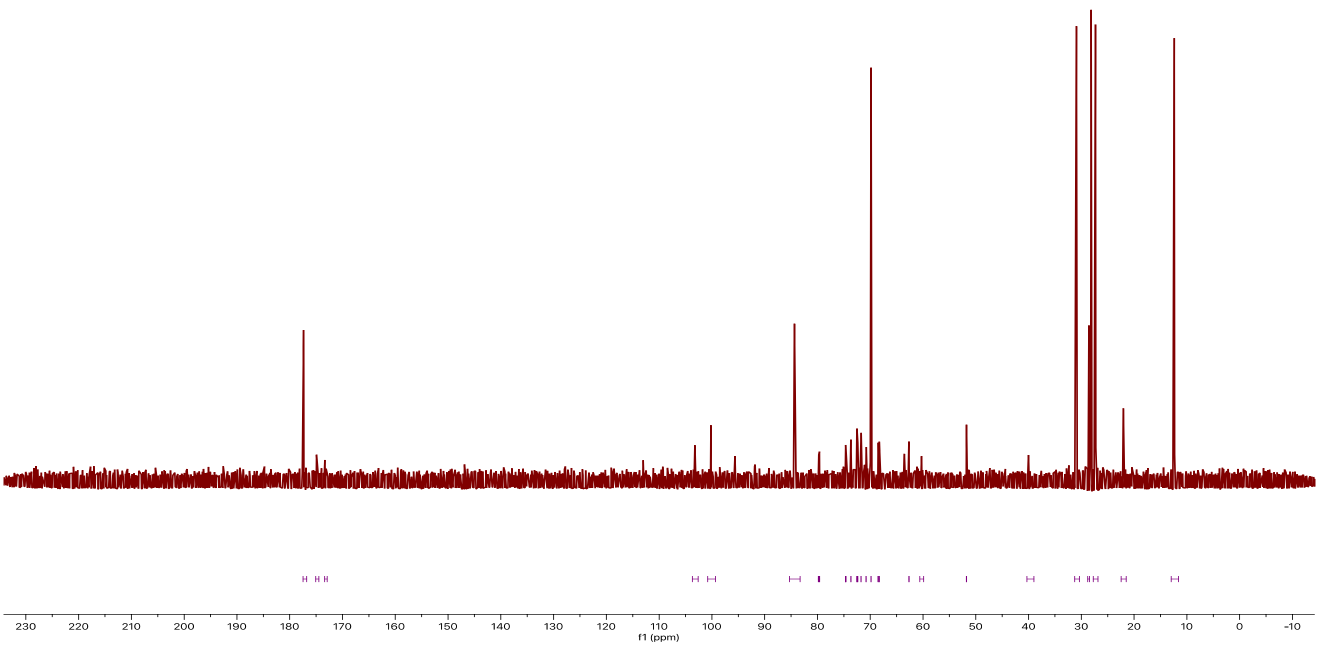

^{1}H NMR (400 MHz, $CDCl_3$) of compound **5**

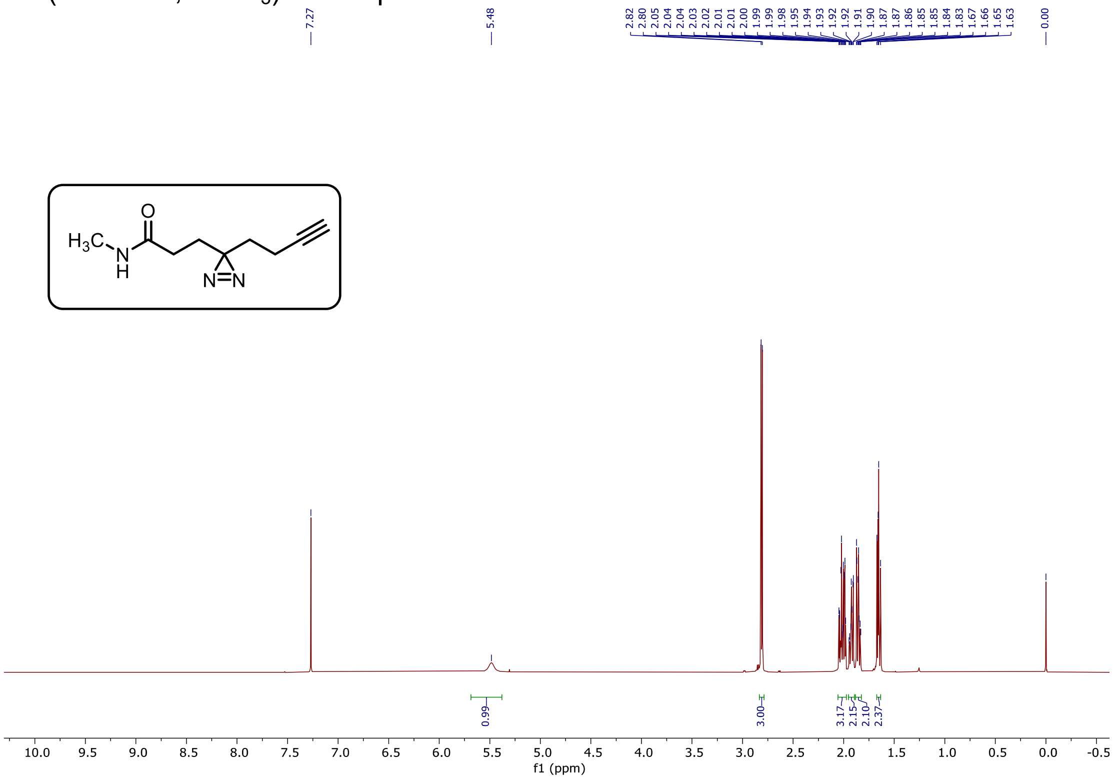

^{13}C NMR (101 MHz, $CDCl_3$) of compound **5**

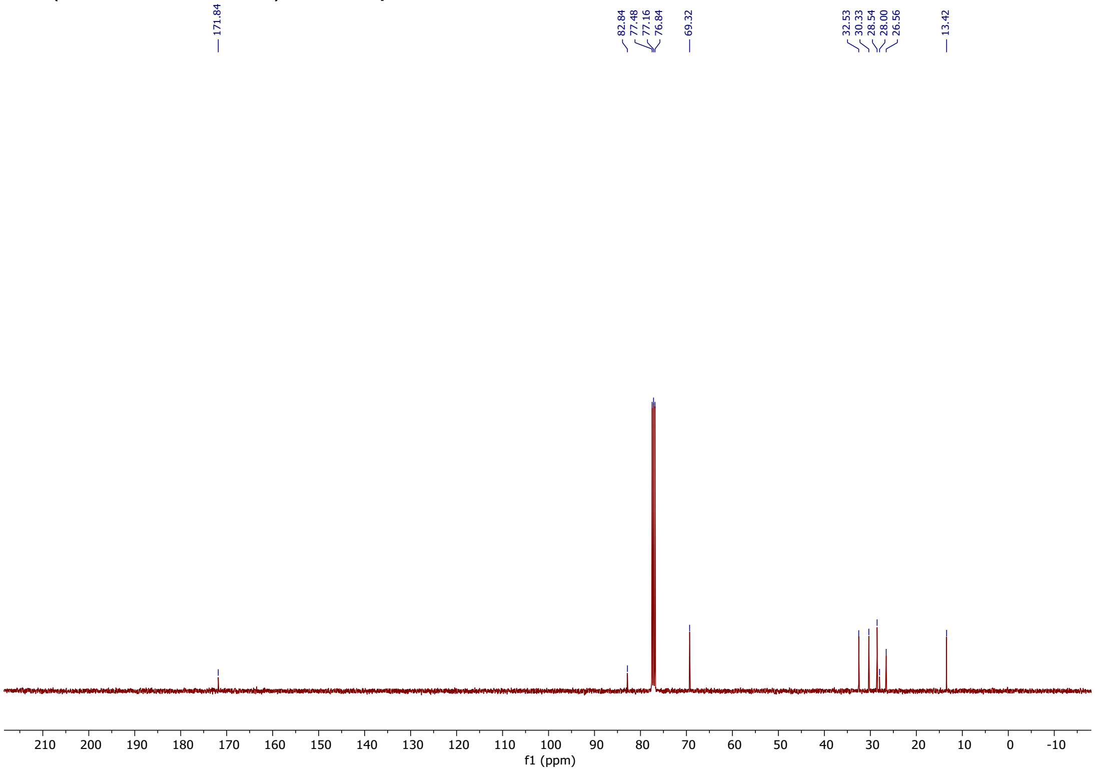

References

1. F. T. Liu, R. J. Patterson and J. L. Wang, *Biochim Biophys Acta*, 2002, **1572**, 263-273.
2. K. C. Haudek, K. J. Spronk, P. G. Voss, R. J. Patterson, J. L. Wang and E. J. Arnoys, *Biochimica Biophysica Acta*, 2010, **1800**, 181-189.
3. M. I. Garin, C. C. Chu, D. Golshayan, E. Cernuda-Morollon, R. Wait and R. I. Lechler, *Blood*, 2007, **109**, 2058-2065.
4. S. A. Chambers and S. D. Townsend, *Carbohyd Res*, 2020, **488**.
5. M. Bejugam and S. L. Flitsch, *Organic Letters*, 2004, **6**, 4001-4004.
6. L. M. Likhosherstov, O. S. Novikova, V. A. Derevitskaja and N. K. Kochetkov, *Carbohyd Res*, 1986, **146**, C1-C5.
7. A. E. Lin, C. A. Autran, A. Szyszka, T. Escajadillo, M. Huang, K. Godula, A. R. Prudden, G. J. Boons, A. L. Lewis, K. S. Doran, V. Nizet and L. Bode, *J Biol Chem*, 2017, **292**, 11243-11249.
8. C. G. Parker, A. Galmozzi, Y. Wang, B. E. Correia, K. Sasaki, C. M. Joslyn, A. S. Kim, C. L. Cavallaro, R. M. Lawrence, S. R. Johnson, I. Narvaiza, E. Saez and B. F. Cravatt, *Cell*, 2017, **168**, 527-541.e529.
9. G. C. McAlister, D. P. Nusinow, M. P. Jedrychowski, M. Wuhr, E. L. Huttlin, B. K. Erickson, R. Rad, W. Haas and S. P. Gygi, *Anal Chem*, 2014, **86**, 7150-7158.
10. J. K. Eng, A. L. McCormack and J. R. Yates, *J Am Soc Mass Spectrom*, 1994, **5**, 976-989.
11. J. E. Elias, W. Haas, B. K. Faherty and S. P. Gygi, *Nat Methods*, 2005, **2**, 667-675.
12. J. E. Elias and S. P. Gygi, *Nat Methods*, 2007, **4**, 207-214.
